# LncRNA SFTA1P promotes cervical cancer progression by interaction with PTBP1 to facilitate TPM4 mRNA degradation

**DOI:** 10.1038/s41419-022-05359-7

**Published:** 2022-11-07

**Authors:** Aoran Luo, Xiaoxiao Lan, Qiongzi Qiu, Qing Zhou, Jia Li, Mengting Wu, Pengyuan Liu, Honghe Zhang, Bingjian Lu, Yan Lu, Weiguo Lu

**Affiliations:** 1 Zhejiang Provincial Key Laboratory of Precision Diagnosis and Therapy for Major Gynecological Diseases, Women’s Hospital and Institute of Translational Medicine, Zhejiang University School of Medicine, Hangzhou, 310006 Zhejiang China; 2 Women’s Reproductive Health Key Laboratory of Zhejiang Province and Department of Gynecologic Oncology, Women’s Hospital, Zhejiang University School of Medicine, Hangzhou, 310006 Zhejiang China; 3 Department of Respiratory Medicine, Sir Run Run Shaw Hospital and Institute of Translational Medicine, Zhejiang University School of Medicine, Hangzhou, 310016 Zhejiang China; 4 Cancer Center, Zhejiang University, Hangzhou, 310013 Zhejiang China; 5 Department of Pathology, Research Unit of Intelligence Classification of Tumor Pathology and Precision Therapy, Chinese Academy of Medical Sciences, Zhejiang University School of Medicine, Hangzhou, 310058 Zhejiang China

**Keywords:** Cervical cancer, Diagnostic markers

## Abstract

Long non-coding RNAs (lncRNAs) play key roles in cancer development and progression. However, the biological function and clinical significance of most lncRNAs in cervical cancer remain elusive. In this study, we explore the function and mechanism of lncRNA surfactant associated 1 (SFTA1P) in cervical cancer. We firstly identified SFTA1P by analyzing the RNA sequencing data of cervical cancer from our previous study and from The Cancer Genome Atlas (TCGA). We then verified SFTA1P expression by qRT-PCR. The cell proliferation and migration capacity of SFTA1P was assessed by using CCK-8, colony formation, transwell and wound healing assays. RNA pull-down, RNA immunoprecipitation (RIP), RNA stability and western blot assays were used to reveal potential mechanisms. Athymic nude mice were used to evaluate tumorigenicity and metastasis in vivo. SFTA1P is upregulated in cervical tumor tissues and its high expression is associated with poor prognosis. Biologically, knockdown of SFTA1P inhibited the proliferation, migration, and invasion of cervical cancer cells in vitro, as well as tumorigenesis and metastasis in vivo. Mechanistically, SFTA1P was shown to interact with polypyrimidine tract binding protein 1 (PTBP1) to regulate the stability of tropomyosin 4 (TPM4) mRNA, thereby resulting in malignant cell phenotypes. TPM4 knockdown could attenuate the suppression of cell progression induced by either SFTA1P or PTBP1 knockdown. Our findings demonstrate that SFTA1P can promote tumor progression by mediating the degradation of TPM4 mRNA through its interaction with PTBP1 protein. This provides a potential therapeutic strategy to target the SFTA1P-PTBP1-TPM4 axis in cervical cancer.

## Introduction

Cervical cancer is the most common gynecologic cancers in women, with 604,127 new cases and 341,831 deaths worldwide each year [1, 2]. Its high prevalence (13.3/100000) and mortality (7.3/100000) impose a heavy burden on public health [1]. Nearly 95% of cervical cancers are caused by persistent infection with high-risk human papillomavirus (HPV) [3]. Although the incidence of cervical cancer recently has declined because of widespread vaccination and screening, cervical cancer still causes a serious threat to women’s reproductive health [4]. Whilst primary surgery with radiotherapy is the main treatment for early stage cervical cancer [5], there remains no effective treatment strategy for advanced metastatic cervical cancer. As a result, cervical cancer still accounts for a significant proportion in cancer-related deaths in women [6]. Therefore, further study of the molecular mechanism of cervical carcinogenesis and progression remains of high priority, particularly towards the exploration of new methods for early diagnosis and treatment.

Long non-coding RNAs (lncRNAs) are a class of functional RNAs over 200 nucleotides in length with little or no protein-coding potential, accounting for a large percentage of non-coding RNAs [7, 8]. Although lncRNAs had been formerly viewed as background noise from junk DNA, accumulating evidence has been more recently suggesting that lncRNAs are involved in various biological processes including differentiation [9], apoptosis [10], inflammation [11] and especially cancer [12]. Numerous studies have also reported that lncRNAs can regulate cellular viability [13], proliferation [12, 14], migration [15, 16], and angiogenesis [17, 18] in cancers. Overall, it has been widely suggested that lncRNA could regulate genes at epigenetic, transcriptional and translational levels [19–21]. Though some studies have begun to fill in some details, the specific molecular mechanisms of lncRNAs in many cancers remain to be further elucidated.

SFTA1P is a novel lncRNA located in Chromosome 10p14 with a full length of 693 bp. Previous studies have demonstrated that SFTA1P is downregulated in lung cancers, with such a downregulation associated with cell migration and invasion [22–24]. In gastric cancer, SFTA1P acts as a tumor suppressor by influencing cell proliferation and migration via down-regulating TP53 [25], while in hepatocellular carcinoma, SFTA1P acts more like an oncogene by down-regulating miR-4766-5p via the PI3K/AKT/mTOR signaling pathway [26]. However, there has been no corresponding studies on SFTA1P in cervical cancer, and any potential roles of SFTA1P in this context remain to be revealed.

In the present study, we verified that lncRNA SFTA1P is overexpressed in cervical cancer tissues and is associated with poor prognosis. In vitro and in vivo functional studies showed that SFTA1P promotes cervical cancer cell proliferation and migration. Analysis of its mechanism revealed that lncRNA SFTA1P regulates cervical cancer progression by interacting with PTBP1 protein to facilitate TPM4 decay. Our findings provide a potential biomarker and therapeutic target for cervical cancer.

## Results

### SFTA1P is highly expressed in cervical cancer tissues and predicts poor prognosis

**Fig. 1:**
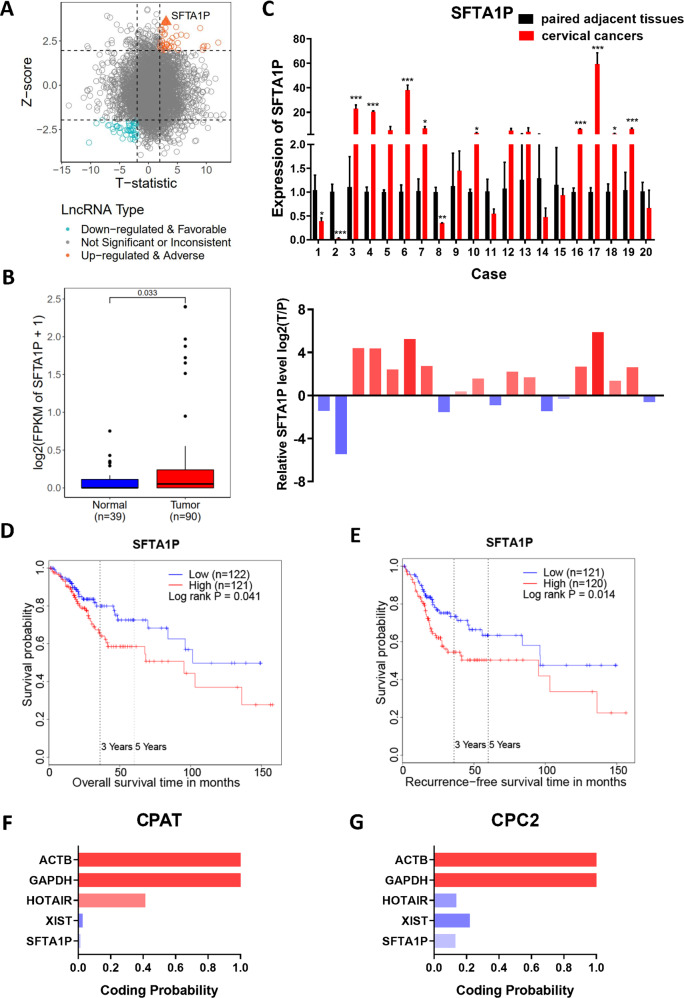
SFTA1P is highly expressed in cervical cancer tissues and predictive of poor prognosis. **A** Scatter plot of 4063 lncRNA expression changes between tumors and adjacent normal tissues (estimated by t-statistic) versus their prognostic value (estimated by z-score). **B** SFTA1P expression in cervical cancer from RNA-seq. **C** SFTA1P expression validated by qRT-PCR in an independent cohort of 20 pairs of cervical cancer tissues and matched adjacent tissues. SFTA1P expression was normalized to the expression of GAPDH. **D**, **E** Kaplan–Meier curves of overall survival and disease-specific survival of cervical cancer patients with low and high expression of SFTA1P. **F**, **G** The protein coding potential of SFTA1P predicted by Coding Potential Assessment Tool (CPAT) and Coding Potential Calculator 2 (CPC2). Data are shown as mean ± SEM. *P < 0.05; **P < 0.01; ***P < 0.001.

To study the potential role of lncRNAs in cervical cancer, we reanalyzed the RNA-seq data of 90 tumors and 39 adjacent normal tissues from patients with cervical cancer from our previous study [27]. We identified 17,082 lncRNAs, 4063 of which were expressed in more than 25% of the samples with an average FPKM of >0.1. These were retained for subsequent differential expression analysis. In these 1912 lncRNAs showed significant differences in expression between tumor and normal tissues (|t-statistics|>1.96, *p*-value < 0.05). We then evaluated the association of these significant lncRNAs with the prognosis of cervical cancer patients, as noted in the The Cancer Genome Atlas (TCGA) database. This analysis yielded 500 lncRNAs associated with overall survival of cervical cancer patients (|z-score|>1.96, *p*-value < 0.05). Forty-six lncRNAs were found to be upregulated in tumors and had a poor prognosis, with SFTA1P showing the most significant association with overall survival (Fig. 1A, B). qRT-PCR analysis of 20 pairs of new cervical cancer and adjacent normal tissues also verified that SFTA1P was highly expressed in cervical cancer tissues in most cases (Fig. 1C). Kapan-Meier survival analysis based on RNA-seq data of cervical cancer from TCGA showed patients with higher expression of SFTA1P had worse prognosis than those with lower expression of SFTA1P (Fig. 1D, E). Use of the Coding Potential Assessment Tool (CPAT) [28] and Coding Potential Calculator 2 (CPC2) [29] further confirmed that SFTA1P is a non-coding RNA with no protein-coding potential (Fig.1 F, G).

### SFTA1P promotes cervical cancer cell proliferation in vitro and in vivo

**Fig. 2:**
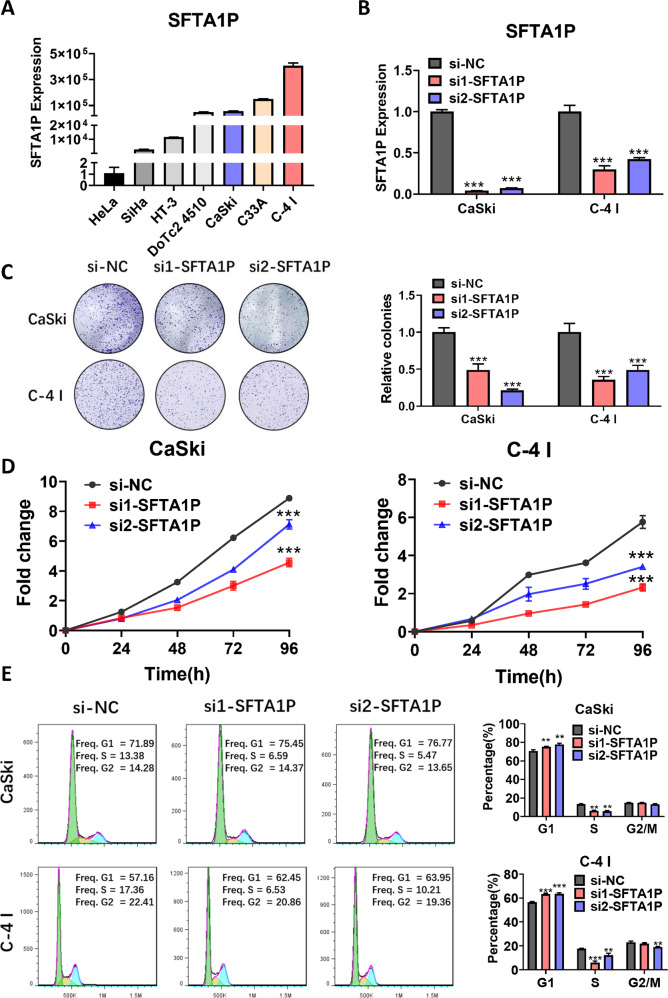
SFTA1P promotes cervical cancer cell proliferation in vitro and in vivo. **A** Relative expression levels of SFTA1P in different cervical cancer cell lines. **B** Relative expression levels of SFTA1P in cervical cancer cells transfected with siRNA, as determined by qRT-PCR. **C** Clonogenic assays of cells transfected with SFTA1P siRNA and negative control (NC). **D** Proliferation of cells transfected with SFTA1P siRNA and NC, as assessed by CCK8 assays. **E** Flow cytometry analysis of cell cycles of cells transfected with SFTA1P siRNA or NC. Data are shown as mean ± SEM. *P < 0.05; **P < 0.01; ***P < 0.001.

To investigate the biological functions of SFTA1P in vitro, we investigated the expression of SFTA1P in seven cervical cancer cell lines. The expression level of SFTA1P was relatively high in CaSki, C33A, and C-4 I (Fig. 2A). Thus we knocked down SFTA1P by transfecting these three cell lines with siRNAs targeting SFTA1P (Fig. 2B and Supplementary Fig.S1A). Knockdown of SFTA1P significantly inhibited cell proliferation in CaSki, C-4 I and C33A cell lines and colony formation in CaSki and C-4 I (Fig. 2C-D and Supplementary Fig. S1B). Conversely, overexpression of SFTA1P significantly promoted cell growth in SiHa cells, but only marginally in HeLa (Supplementary Fig. S1C-D). In addition, SFTA1P knockdown could increase the G0/G1 cell proportion and decrease the S phase cell proportion compared with the controls, indicating that depletion of SFTA1P causes G1 arrest (Fig. 2E). To further examine the function of SFTA1P in vivo, we subcutaneously injected CaSki or C-4 I cells stably knocked down SFTA1P into the flanks of nude mice. Nude mice in the Sh-SFTA1P (SFTA1P knockdown) group had significantly smaller tumor volume and weight than the control group (Supplementary Fig. S1E), suggesting SFTA1P as a promoter of tumorigenicity of cervical cancer cells in vivo.

### SFTA1P promotes cervical cancer cells metastasis in vitro and in vivo

**Fig. 3:**
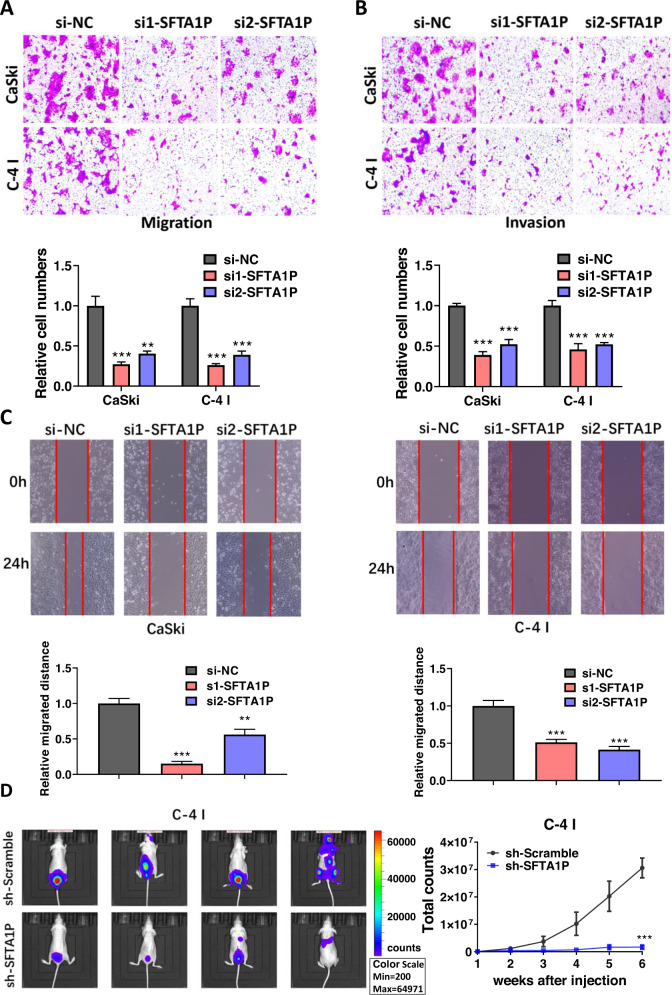
SFTA1P promotes cervical cancer cell metastasis in vitro and in vivo. **A**, **B** Migration and invasion ability of CaSki and C-4 I cells transfected with SFTA1P siRNA or NC. **C** Wound healing assays of CaSki and C-4 I cells transfected with SFTA1P siRNA or NC. **D** SFTA1P promotes cervical cancer cell metastasis in vivo. Nude mice were injected intravenously with sh-scramble or sh-SFTA1P cells via the tail vein. Secondary metastatic sites were observed mainly in the pelvic cavity, followed by the lung and brain. The mice were imaged weekly for 6 weeks. Data are shown as mean ± SEM. *P < 0.05; **P < 0.01; ***P < 0.001.

To explore the metastatic ability of SFTA1P in cervical cancer cells, we carried out migration and invasion assays. SFTA1P knockdown significantly reduced migration and invasion ability of CaSki and C-4 I cells (Fig. 3A, B). While SFTA1P overexpression could promote cell migration of SiHa and Hela cells (Supplementary Fig. S1F). Wound healing assays were also used to verify the function of SFTA1P knockdown in cervical cancer cells and the results showed the relative migration distances were decreased in the si-SFTA1P group as compared with the control group (Fig. 3C). To further evaluate the effect of SFTA1P on tumor invasion in vivo, athymic nude mice were injected intravenously with C-4 I cells stably transfected with sh-scramble or sh-SFTA1P via the tail vein. It was confirmed that knockdown of SFTA1P could reduce in vivo metastasis of cervical cancer cells (Fig. 3D).

### SFTA1P interacts with PTBP1

**Fig. 4:**
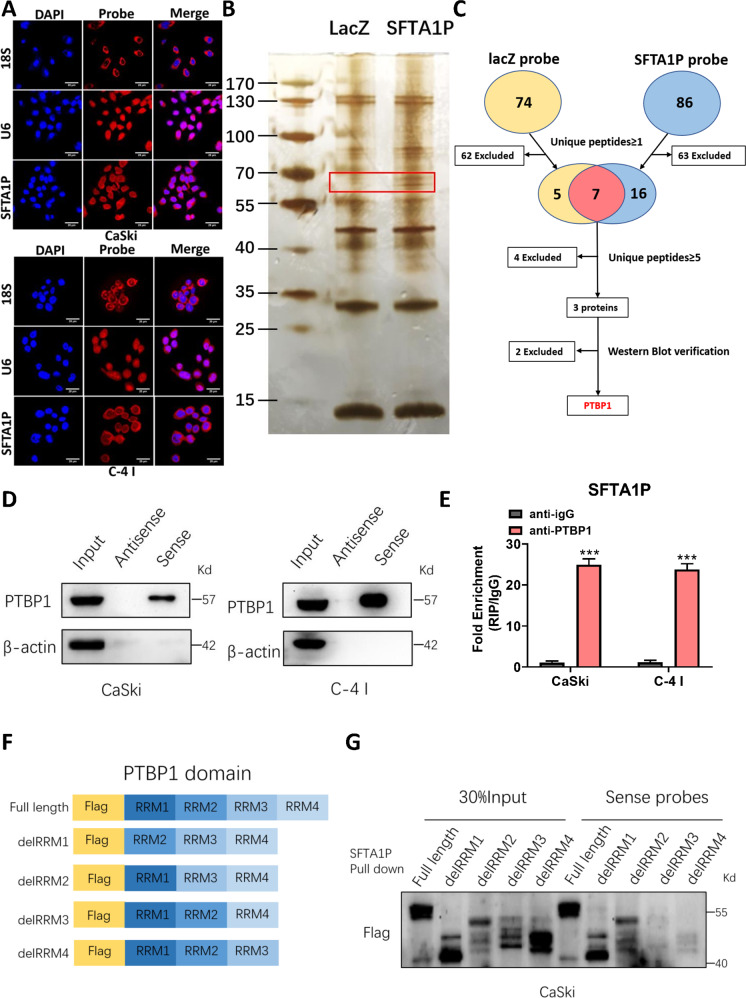
SFTA1P interacts with PTBP1 in cervical cancer cells. **A** The localization of SFTA1P in the cytoplasm of CaSki and C-4 I cells by RNA-FISH assays. Scale bar, 20 μM. **B** Potential proteins pulled down by SFTA1P or lacZ probes in C-4 I cells. The red box indicates distinct protein bands between SFTA1P or lacZ probes. **C** Flowchart for identifying proteins that interact with SFTA1P. **D** Western blot analysis of PTBP1 in sense and antisense SFTA1P pull-down fractions from CaSki and C-4 I cells. **E** RNA immunoprecipitation (RIP) assay with antibody PTBP1 in CaSki and C-4 I cells. The relative fold enrichment of SFTA1P between PTBP1 and IgG RIP fractions was measured by qRT-PCR. **F** The schematic domain structure of PTBP1 and four domain deleted mutants. **G** Truncated RRM domains were detected by RNA pull-down, followed by western blot confirmation. Data are shown as mean ± SEM. *P < 0.05; **P < 0.01; ***P < 0.001.

To explore the underlying mechanism of SFTA1P, RNA-FISH was first used to determine the subcellular localization of SFTA1P in cervical cancer cells. SFTA1P was mainly localized in the cytoplasm of CaSki and C-4 I cells (Fig. 4A), indicating that SFTA1P may regulate target protein expression at the posttranscriptional level by sponging microRNAs or modulating RBPs [30]. Then, we performed RNA pull down assays in C-4 I cells to identify potential SFTA1P-interacting proteins. Distinct bands between control lacZ and SFTA1P sense, with weights between 55 and 70 kDa, were excised from the gel and then subjected to mass spectrometry analysis (Fig. 4B). Proteins with incorrect molecular weights or non-specifically bound proteins on control lacZ and SFTA1P sense were excluded. PTBP1 was selected as one of the top candidates for follow-up research (Fig. 4C and Supplementary Fig. S2B, C). The physical interaction between SFTA1P and PTBP1 was further validated by western blot with SFTA1P antisense and sense (Fig. 4D) and RIP analysis with PTBP1 antibodies (Fig. 4E). This was consistent with the prediction of the RBPmap database (http://rbpmap.technion.ac.il/) predicting that SFTA1P may bind to PTBP1 (Supplementary Fig. S2A). PTBP1 is an RNA-binding protein with 4 RNA recognition motifs (RRMs) according to Uniprot (https://www.uniprot.org/). To determine structural determinants of the interactions between SFTA1P and PTBP1, we carried out deletion mapping of PTBP1 functional domains. After transfecting plasmids with Flag tag, we examined their ability to bind to SFTA1P by RNA pull-down assay, followed by Flag protein immunoblotting analysis. The interaction of SFTA1P and PTBP1 with either RRM3 domain or RRM4 domain deletion was decreased (Fig. 4F, G), suggesting these two domains may be key structures for PTBP1 to bind SFTA1P.

### TPM4 is a candidate downstream gene of SFTA1P and PTBP1

**Fig. 5:**
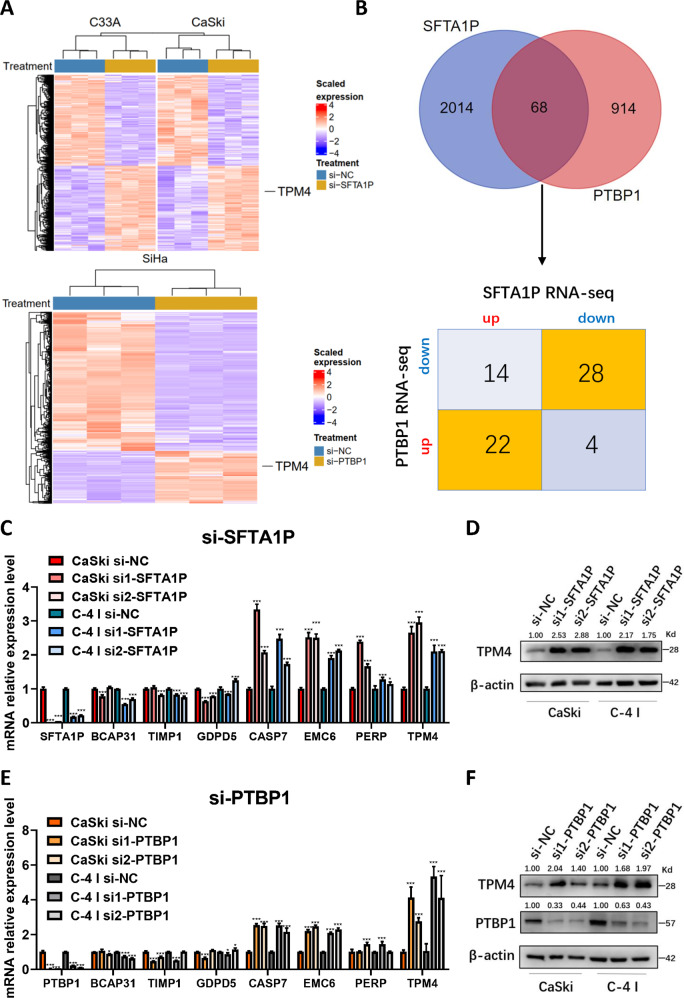
TPM4 is a downstream gene of SFTA1P and PTBP1 in cervical cancer cells. **A** Heatmap of differentially expressed genes upon knockdown of SFTA1P in C33A and CaSki and knockdown of PTBP1 in SiHa cells, revealed by RNA-seq. **B** Venn diagrams of differentially expressed genes (DEGs) identified by SFTA1P RNA-seq and PTBP1 RNA-seq (upper) and intersecting DEGs after knockdown of SFTA1P and PTBP1 (lower). **C** Eight key candidate downstream genes were selected based on our RNA-seq data and literature review, and subsequently evaluated by RT-PCR in CaSki and C-4 I cells transfected with SFTA1P siRNA. **D** Western blot analysis of TPM4 in CaSki and C-4 I cells with SFTA1P knockdown. **E** Eight key candidate downstream genes were evaluated by RT-PCR in CaSki and C-4 I cells transfected with PTBP1 siRNA. **F** Western blot analysis of TPM4 in cervical cancer cells with PTBP1 knockdown. Data are shown as mean ± SEM. *P < 0.05; **P < 0.01; ***P < 0.001.

It has been previously reported that PTBP1 plays a tumor-promoting role in cancer progression [31–33] and is associated with cervical lesion progression and carcinogenesis in our former studies [34, 35]. To find related downstream genes, we performed RNA-seq on SFTA1P knockdown cells (upper panel in Fig. 5A) and simultaneously analyzed PTBP1 RNA-seq data downloaded from the GEO database (GSE168907) (lower panel in Fig. 5A). The Venn diagram shows that there were 68 genes co-regulated by SFTA1P and PTBP1 (Fig. 5B). Considering that SFTA1P and PTBP1 have the same tumor-promoting roles in cervical cancer, we focused on genes that were simultaneously up- or down-regulated in SFTA1P knockdown and PTBP knockdown cancer cells. We examined several genes by qPCR and western blot analyses and showed that SFTA1P and PTBP1 knockdown could consistently increase both mRNA and protein levels of TPM4 to a greater extent, suggesting that TPM4 is regulated by SFTA1P and PTBP1. According to our previous RNA-seq data [27], CASP7, EMC6 and PERP were upregulated in cervical tumor tissues, which seemed to conflict with the qPCR results of SFTA1P knockdown (Supplementary Fig. S3A). Therefore these three genes were excluded. BCAP31 was also excluded as it was not significantly different in western blots between si-PTBP1 and control groups (Supplementary Fig. S3B, C). Taken together, we selected TPM4 as the main downstream candidate of SFTA1P and PTBP1 for follow-up studies.

### TPM4 inhibits cervical cancer cell proliferation and metastasis in vitro

**Fig. 6:**
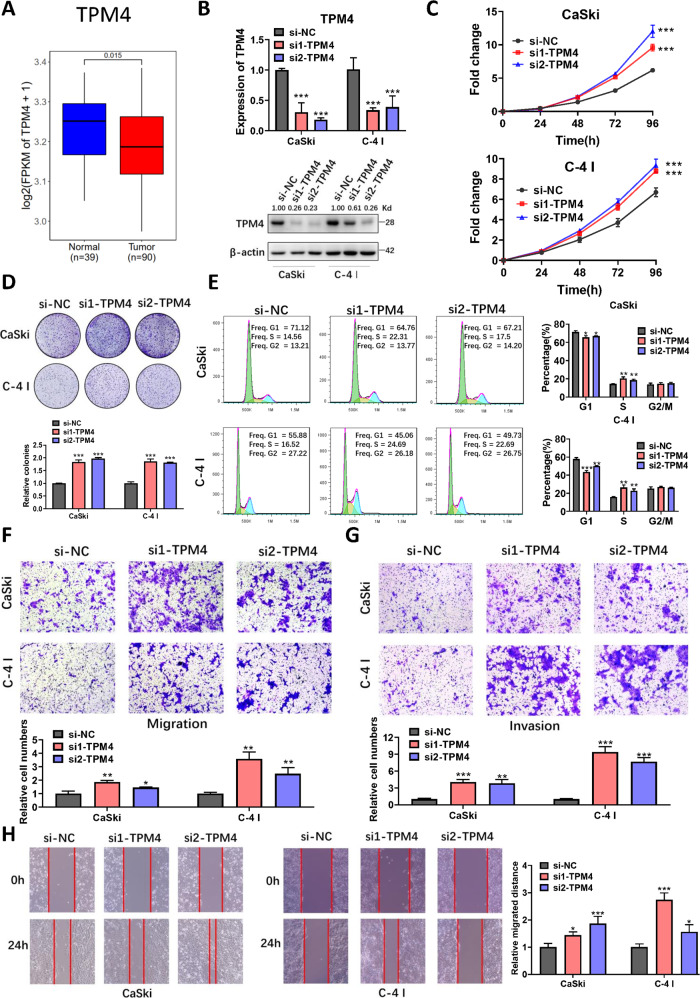
TPM4 inhibits cervical cancer cell proliferation, migration, and invasion in vitro. **A** TPM4 expression in cervical cancer from RNA-seq data. **B** Relative expression of TPM4 in cervical cancer cells transfected with siRNA as determined by qRT-PCR and western blot. **C** Proliferation of cervical cancer cells transfected with TPM4 siRNA as assessed by CCK8 assay. **D** Clonogenic assays of cervical cancer cells transfected with TPM4 siRNA. **E** Flow cytometry analysis of cell cycles of cells transfected with TPM4 siRNA. **F**, **G** Migration and invasion of cervical cancer cells transfected with TPM4 siRNA, assessed by transwell assays. **H** Wound healing assays of cervical cancer cells transfected with TPM4 siRNA. Data are shown as mean ± SEM. *P < 0.05; **P < 0.01; ***P < 0.001.

Whilst previous studies have reached contradictory conclusions as to whether TPM4 acts as either an oncogene or anti-oncogene in human cancers [36–39], its role in cervical cancer remains unclear. Our RNA-seq data showed that TPM4 is down‐regulated in cervical cancer tissues (Fig. 6A). To investigate the biological functions of TPM4 in cervical cancer, we detected the expression of TPM4 in cervical cancer cell lines and knocked down TPM4 using siRNAs in CaSki and C-4 I cells with relatively high basal TPM4 expression (Fig. 6B and Supplementary Fig. S4A). CCK8 and colony formation indicated that TPM4 knockdown significantly promoted proliferation in cervical cancer cells (Fig. 6C, D). In contrast to SFTA1P knockdown, TPM4 knockdown induced a decrease in the proportion of cells in the G1-phase accompanied by a corresponding increase in the S-phase (Fig. 6E). Results from transwell assays and wound healing also demonstrated that TPM4 knockdown promoted the migration and invasion of cervical cancer cells (Fig. 6F–H). Taken together, these results imply that TPM4 may act as a tumor suppressor in cervical cancer cells.

### SFTA1P and PTBP1 promote the progression of cervical cancer cells by regulating TPM4

**Fig. 7:**
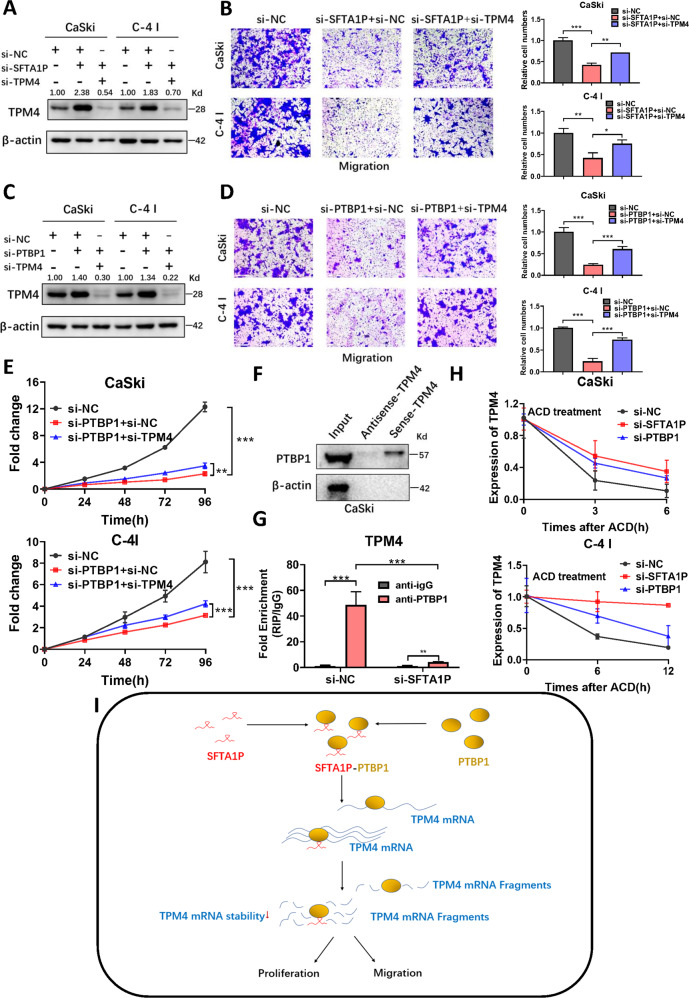
SFTA1P and PTBP1 promote the malignant process of cervical cancer cells by regulating TPM4. **A** Western blot analysis of TPM4 in cervical cancer cells with knockdown of SFTA1P and TPM4. **B** Migration of cervical cancer cells transfected with SFTA1P and TPM4 siRNAs. **C** Western blot analysis of TPM4 in cervical cancer cells with knockdown of PTBP1 and TPM4. **D** Migration of cervical cancer cells transfected with PTBP1 and TPM4 siRNAs. **E** Proliferation of cervical cancer cells transfected with PTBP1 and TPM4 siRNAs, assessed by CCK8 assays. **F** Western blot analysis of PTBP1 in sense and antisense TPM4 pull-down fractions from CaSki cells. **G** RIP assays with anti-PTBP1 or IgG in CaSki cells transfected with SFTA1P siRNA or NC. The relative fold enrichment of TPM4 between PTBP1 and IgG RIP fractions was measured by qRT-PCR. **H** CaSki and C-4 I cells transfected with SFTA1P or PTBP1 siRNA were treated with actinomycin D (5 μg/ml) at various time points. RNA was extracted at different time points and TPM4 mRNA was analyzed by qPCR and normalized to GAPDH. **I** Proposed model of the SFTA1P-PTBP1-TPM4 axis regulating the proliferation, migration, and invasion of cervical cancer cells. SFTA1P forms a complex with PTBP1, which can promote the malignant progression of cervical cancer by facilitating the binding of PTBP1 to TPM4 mRNA and thus increasing its degradation. Data are shown as mean ± SEM. *P < 0.05; **P < 0.01; ***P < 0.001.

To investigate whether SFTA1P mediates the function of TPM4 in cervical cancer cells, we co-transfected si-SFTA1P and si-TPM4 in CaSki and C-4 I cells (Fig. 7A). Decreased migration induced by SFTA1P knockdown was rescued by co-knockdown of SFTA1P and TPM4 (Fig. 7B). Similarly, we also explored the role of PTBP1 in regulating the function of TPM4 in cervical cancer. Results indicated that the reductions in cell proliferation and migration induced by PTBP1 knockdown was rescued by the co-knockdown of PTBP1 and TPM4 (Fig. 7C–E). Previous studies have shown that PTBP1 can bind to target mRNAs and influence their stability [40, 41]. Therefore, we explored whether PTBP1 could bind to TPM4 mRNA. RBPmap predicted that TPM4 mRNA may contain potential PTBP1 binding sites within its 3’UTR (Supplementary Fig. S5). RNA pull-down and RIP assays also verified binding between PTBP1 and TPM4 mRNA (Fig. 7F, G). It was also demonstrated that SFTA1P knockdown significantly reduced the enrichment of TPM4 mRNA by PTBP1 (Fig. 7G). Finally, an additional RNA stability experiment showed that either SFTA1P or PTBP1 knockdown led to increased TPM4 mRNA stability (Fig. 7H). All of the above suggested that SFTA1P and PTBP1 promote the malignant process of cervical cancer cells by regulating TPM4 mRNA stability.

## Discussion

Over recent years, lncRNAs have received increasing attention due to their biological functions in various diseases. Among these, it has been reported that lncRNAs play a critical role in various cancers, such as lung, breast and colorectal cancer [12, 42, 43]. Previous studies have showed that whilst LncRNA SFTA1P is up-regulated in hepatocellular carcinomas it is down-regulated in lung carcinoma and gastric cancer [24–26]. The present study revealed that SFTA1P is upregulated in cervical cancer tissues, its higher expression being highly predictive of worse prognosis. We further demonstrated that SFTA1P acts as an oncogene by promoting cervical cancer cell proliferation, migration, and invasion, both in vitro and in vivo.

Increasing evidence suggests that the function of lncRNAs is closely related to their subcellular localization [30]. Our FISH assay demonstrated that SFTA1P is mainly localized in the cytoplasm, indicating that SFTA1P may regulate target protein expression at a posttranscriptional level by sponging microRNAs or modulating RBPs. Thus, we utilized RNA pull-down and mass spectrometry analysis to identify potential proteins interacting with SFTA1P, with PTBP1 being the lead candidate. An RIP assay further verified the physical interaction between SFTA1P and PTBP1. PTBP1 is a member of the heterogeneous nuclear ribonucleoprotein (hnRNP) family, which is involved in splicing regulation, IRES-mediated translation initiation, and mRNA stability [13, 40, 41, 44]. It has been reported to be up-regulated to exert tumor-promoting roles in a variety of cancers including cervical cancer [31–35]. However, the biological functions of PTBP1 in cervical cancer remains to be explored. Considering that SFTA1P and PTBP1 share the same tumor-promoting effect, we focused on genes that were simultaneously up- or down-regulated in SFTA1P RNA-seq and PTBP1 RNA-seq experiments. By qPCR and western blot analyses, we found that TPM4 was the most likely downstream gene of the SFTA1P-PTBP1 complex.

Although there are a few studies on the biological function of TPM4 in cancers [36–39], the role of TPM4 in cervical cancer remains unclear. In the present study, we demonstrated that TPM4 knockdown can promote the proliferation and migration of cervical cancer cells, suggesting that TPM4 acts as a tumor suppressor in cervical cancer. Furthermore, TPM4 knockdown rescued the reductions of malignant phenotypes induced by SFTA1P and PTBP1, suggesting that SFTA1P and PTBP1 may function through TPM4.

We then explored the underlying mechanism by which SFTA1P and PTBP1 influence TPM4 in cervical carcinogenesis and progression. PTBP1 is a canonical RNA-binding protein which has many functions including alternative splicing, mRNA stability and polyadenylation [45]. We thus investigated the role of SFTA1P and PTBP1 in regulating TPM4 mRNA. RNA pull down and RIP assays showed that PTBP1 could bind with TPM4 mRNA, which was attenuated by SFTA1P knockdown. Knockdown of SFTA1P and PTBP1 increases the stability of TPM4 mRNA, suggesting that PTBP1 can promote the degradation of TPM4 mRNA, and that this degradation will be enhanced in the presence of SFTA1P.

Finally, there are some limitations in this study. Whether PTBP1 can act on TPM4 through its other functions, such as alternative splicing, remains unanswered. The clinical therapeutic potential of the SFTA1P-PTBP1-TPM4 axis in cervical cancer also awaits further investigation.

In conclusion, the lncRNA SFTA1P is up-regulated and associated with poor prognosis in cervical cancer. SFTA1P can promote cervical carcinogenesis and progression by regulating PTBP1 protein-mediated degradation of TPM4 mRNA. These findings provide a potential therapeutic strategy to target the SFTA1P-PTBP1-TPM4 axis in cases of cervical cancer.

## Materials and methods

### Human cervical cancer samples

To evaluate the expression of SFTA1P, 20 paired cervical cancer tissue samples, with corresponding adjacent normal tissues from the surgical specimen archives of Women’s Hospital of Zhejiang University School of Medicine (Hangzhou, China) were obtained at the time of diagnosis and prior to the initiation of any treatment. RNA was extracted from snap-frozen tissue specimens stored at −80 °C in liquid nitrogen. The diagnosis was confirmed by reviewing H&E slides by a gynecologic pathologist. Research involving human subjects was conducted in accordance with the International Ethical Guidelines for Biomedical Research. All subjects participating in the study provided informed consent.

### Identification of LncRNA using RNA-Seq data

RNA-seq data of 90 tumors and 39 adjacent normal tissues from patients with cervical cancer were obtained from our previous study [27]. After read alignment, transcript assembly and quantification, as previously described, differentially expressed genes and lncRNAs between tumors and adjacent normal tissues were calculated using a Student’s t-test. The Bonferroni correction was used to adjust p-values.

Our previous workflow with parameter tuning was followed for the prediction of lncRNAs [46]. Briefly, transcripts with multi exons and >160 bp in length were kept for downstream analysis. PhyloCSF was used to access the protein coding potential of the remaining transcripts by aligning them to genomes from multiple species, including chimpanzee, rhesus monkey, mouse, guinea, pig, cow, horse and dog [47]. Transcripts that met any of the following criteria were discarded: PhyloCSF score >50, complete branch length (CBL) > 0, open reading frame (ORF) > 150 amino acids, or CBL = 0 but OFR > 50. Finally, transcripts with a median E-value greater than 1e−18 by blastx were retained as candidate lncRNAs.

### Survival analysis

RNA-Seq data and clinical data of cervical cancers from TCGA were downloaded from the GDC Data Portal (https://portal.gdc.cancer.gov/) as described in our previous study [27]. The association of each lncRNA with overall survival was calculated using a univariate Cox proportional hazards model. P-values, hazard ratios with a 95% confidence interval and z-scores were calculated. Survival analyses were performed using the R package “survival”.

### Cell culture

Cervical cancer cell lines C33A, CaSki, C-4 I, SiHa, DOTC2 4510 and HT-3 were purchased from ATCC (Supplementary Figs. S6–S8). HeLa was a gift from other lab and authenticated by STR typing (Supplementary Fig. S9). CaSki was cultured in 1640, C-4 I was in Waymouth’s MB 752/1, other cell lines were in MEM medium, respectively, containing 10% FBS, 100 ng/mL streptomycin, 100 U/mL penicillin and 2 umol/mL in 5% CO_2_, in a 37 °C cell incubator. The cells were subcultured when the degree of fusion reached 80–90%.

### RNA extraction and RT-qPCR

RNA extraction and RT-qPCR were conducted for each sample as previously described [34, 48]. All primers are listed in Supplementary Table S1.

### Cell transfection

The C33A, CaSki and C-4 I cells were cultured to 50% confluence in 6-well plates and were transfected by using transfection reagents (SignaGen, Frederick, MD) according to the manufacturer’s instructions. The silencing effect of siRNA interference was detected 24 h after transfection.

### Proliferation assays

C33A (4000 cells/well), CaSki (2000cells/well), and C-4 I(4000cells/well) were transfected with siRNAs and then were plated in a 96-well plate. Cell Counting Kit-8 (CCK-8) (Dojindo, Tokyo, Japan) was used to measure cell proliferation at 24, 48, 72 and 96 h after transfection. The absorbance at 450 nm was measured by microplate reader.

### Colony formation assays

Colony formation assays were performed to monitor the clonality of cervical cancer cells. Treated CaSki (1000 cells/well) and C-4 I (2000/well) cells were seeded into 6-well plates and cultured for 10 days. Colonies were stained with crystal violet after formaldehyde fixation. The number of visible colonies was counted by ImageJ software (https://imagej.net/). Each experiment was repeated three times.

### Migration and invasion assays

Cell migration and invasion assays were carried out using 24-well transwell plates (Corning Costar, Tewksbury, MA, USA). Cervical cancer cells were transfected with siRNA or negative control for 24 h and then starved for 24 h with serum free medium. 1 × 10^5^ cells for CaSki and 1.5 × 10^5^ cells for C-4 I were plated with 300 μL serum-free media into uncoated or matrigel-coated upper chamber for migration or invasion assay. The lower chambers were filled with medium supplemented with 20% FBS. Plates were incubated in 5% CO2 at 37 °C overnight. Each membrane was photographed and migratory cells were counted under a microscope.

### Wound healing assays

The wound healing assays were performed by using culture-inserts (Ibidi GmbH, Munich, Germany) as described previously [34]. Cells were seeded in 70 μL medium at a density of 7 × 10^5^ cells/mL (CaSki) and 10 × 10^5^ cells/mL (C-4 I) on each side of the culture-inserts, into 12-well plate. The inserts were removed after 24 h, and the cells were washed with phosphate-buffered saline (PBS). The wound healing ratio was determined by collecting images at the indicated time points.

### Cell cycle analysis

Cell cycle progression was determined using a Cell Cycle and Apoptosis Analysis Kit (C1052, Beyotime, Shanghai, China) according to the manufacturer’s instructions. The stained cells were analyzed by flow cytometry (Beckman Coulter Cytoflex, Beckman, USA).

### Western blot assays

Cells were lysed using RIPA buffer (Beyotime) containing protease and phosphatase inhibitors. Cellular lysates were centrifuged at 12,000 rpm for 20 min and then denatured in boiling water for 10 min. Total proteins were separated using sodium dodecyl surfate-polyacrylamide gel electrophoresis (SDS-PAGE) and transferred onto polyvinylidene fluoride (PVDF) membranes. Incubation with antibodies was performed after blocking the membrane with 5% skim milk. The antibodies used were as follows:β-actin, Flag, PTBP1, TPM4, BCAP31 and MCCC2 (Supplementary Table S2).

### In vivo xenograft model

For the in vivo tumorigenicity assay, 4–5 weeks old female BALB/c nude mice were randomly divided into two groups. CaSki and C-4 I cells stably transfected with sh-scramble or sh-SFTA1P were dissociated using trypsin and washed twice with sterilized PBS. Then, 100 μL of PBS containing 2 × 10^6^ cells was subcutaneously inoculated into the flank of mice. Tumor growth was measured after 6 days of tumor implantation. Two (CaSki) or three (C-4 I) weeks after inoculation, the mice were sacrificed according to the policy for treating tumor-bearing animals humanely. For the in vivo invasion assay, 2 × 10^6^ C-4 I cells stably transfected with sh-scramble or sh-SFTA1P were injected intravenously into the tail vein of nude mice. C-4 I cells stably transfected with sh-scramble or sh-SFTA1P were injected intravenously into the tail vein of nude mice. Luciferin (Gold Biotech, St Louis, MO, USA) was administered weekly to the mice by intraperitoneal injection. Twenty minutes after each administration, the mice were imaged using IVIS@ Lumina II system (Caliper Life Sciences, Hopkinton, MA, USA). All experiments were performed in accordance with the Guide for the Care and Use of Laboratory Animals (NIH publication 80–23, revised 1996) and the approval of the Zhejiang University animal ethics committee.

### Biotin-labeled RNA pull-down assay and mass spectrometry analysis

The RNA pull-down assays were performed using a Pierce™ Magnetic RNA-Protein Pull-down Kit (Thermo Scientific, Irwindale, CA, USA, Catalog # 20164) [49]. Briefly, biotin-labeled DNA probes including anti-sense and sense probes were incubated with streptavidin magnetic beads for 3 h at room temperature. The lysates of the cells were incubated overnight at 4 °C with streptavidin magnetic beads. Proteins bound to magnetic beads were separated using SurePAGE and excised for mass spectrometry analysis (Lumingbio, Shanghai, China).

### RNA immunoprecipitation (RIP) assay

RIP assay was implemented with a Magna RNA immunoprecipitation kit (Millipore, Bedford, MA, USA) according to the manufacturer’s instructions. Briefly, cell suspension was prepared in RIP buffer. Cell suspensions were incubated overnight at 4 °C with anti-PTBP1 antibody (Abcam). After precipitation, RNA was purified and analyzed by qRT-PCR.

### RNA sequencing

CaSki cells and C33A cells transfected with si-NC or si-SFTA1P were cultured for 48 h after transfection. Each group prepared three independent assay samples. The total RNA was extracted using Trizol (Invitrogen). A TruSeq RNA Sample Prep Kit (Illumina) was used to prepare DNA libraries. To ensure uniform cluster density, Illumina’s qPCR quantification guide was used to quantify libraries. RNA-seq data of SFTA1P knockdown (Supplementary Table S3) was aligned to the human genome (hg19) using TopHat2 (v 2.0.13) [50]. Transcripts were assembled from RNA-seq alignments using Stringtie2 (v2.1.0) [51]. An evaluation of gene expression was based on the fraction of fragments per kilobase of transcript per million reads mapped (FPKM). Gene expression differences between knockdown and control cells were detected using a linear model with cell line as a covariate using the R statistical package.

### RNA stability

Cells were transfected with siRNA or negative control for 48 h and added actinomycin-D (5 μg/ml) to block mRNA synthesis. RT-qPCR analysis of total RNA was carried out at different time points. The relative abundance of TPM4 mRNA was calculated using the ΔΔCt method and normalized to GAPDH. At 0 h following actinomycin D treatment, mRNA was arbitrarily set to 1.

### Statistical analysis

Data are expressed as the mean ± SD. Differences between groups were examined by either Student’s *t* test or one- or two-way ANOVA. Statistical significance was defined as *p* < 0.05. ^∗^*p* < 0.05, ^∗∗^*p* < 0.01, ^∗∗∗^*p* < 0.001. Data analyses were carried out using the GraphPad Prism 8.0 (GraphPad Software).

## Supplementary information

SFTA1P-Supplementary Materials

Original Data File

Reproducibility checklist

## Data Availability

Data supporting this study are available from the corresponding authors upon reasonable request.
