# Supplementary material for: LncRNA SFTA1P promotes cervical cancer progression by interaction with PTBP1 to facilitate TPM4 mRNA degradation
Source: Cell Death Dis. 2022 Nov 7;13(11):936. doi: 10.1038/s41419-022-05359-7 (PMC9640654; doi:10.1038/s41419-022-05359-7)
Supplement: Supplementary file 1 — SFTA1P-Supplementary Materials [file 41419_2022_5359_MOESM1_ESM.pdf]

LncRNA SFTA1P promotes cervical cancer progression by interaction with PTBP1 to facilitate TPM4 mRNA degradation

Supplementary Materials

Table S1 Primers and probes used in this study

Target	Primers/siRNAs/shRNAs/probes	Sequence(5'→3')
SFTA1P	Forward primer	CAGCATTCCAGGACTCCTCA
	Reverse primer	TCAAGGGCAATATTCCGGGT
GAPDH	Forward primer	TTCACCACCATGGAGAAGGC
	Reverse primer	GGCATGGACTGTGGTCATGA
BCAP31	Forward primer	CGGCTGGTGGAGTTGTTAGT
	Reverse primer	CGGGATTGTTCTGGAGGTT
TIMP1	Forward primer	TTTTGTGGCTCCCTGGAACA
	Reverse primer	GGATAAACAGGGAAACACTGTGC
GDPD5	Forward primer	TCTTCGTGCTCCAGAAGTGG
	Reverse primer	AAGCTTCTCCTTCATGATGCT
CASP7	Forward primer	GTGGGAACGATGGCAGATGA
	Reverse primer	GAGGGACGGTACAAACGAGG
EMC6	Forward primer	GCCGCCGTCCTGGATTATT
	Reverse primer	GAGGCGAGCAGGTAGAAGAT
PERP	Forward primer	GCATGAAGGGTGAAGGTCTG
	Reverse primer	GATGCTTGTCTTCCTGAGAGTG
TPM4	Forward primer	ACGGTTGCAAAACTGGAAAA
	Reverse primer	TTGGCTCTGGATGGAAAATC
NC	siRNA	UUCUCCGAACGUGUCACGUTT
SFTA1P	si1RNA	GUCAACACCUGCUCAUAGATT
	si2RNA	GAGGAGAUUAGCUGCUAGATT
PTBP1	si1RNA	CCCUCAUUGACCUGCACAATT
	si2RNA	GCACAGUGUUGAAGAUCAUTT
TPM4	si1RNA	CCGCAAAUACGAGGAGGUATT
	si2RNA	GGAUCAGACACUAAACGAATT
Scramble	shRNA	ccggTTCTCCGAACGTGTCACGTctcgag<br>ACGTGACACGTTCGGAGAAtttttg
SFTA1P	shRNA	ccggGTCAACACCTGCTCATAGActcga<br>gTCTATGAGCAGGTGTTGACtttttg
lacZ	probe1	TTAAAGCGAGTGGCAACATG-biotin
	probe2	TCACGACGTTGTAAAACGAC-biotin
SFTA1P	sense1	ACCTGGAATGCTGTATAACC-biotin
	sense2	ATTGATGGTGAATGCCTTTC-biotin
	antisense1	GGTTATACAGCATTCCAGGT-biotin
	antisense2	GAAAGGCATTCACCATCAAT-biotin
TPM4	sense1	CTCAAAGCTTCTCCGATACA-biotin
	sense2	CTTACTAGGAAATACCCTGC-biotin
	antisense1	TGTATCGGAGAAGCTTTGAG-biotin
	antisense2	GCAGGGTATTTCCTAGTAAG-biotin

Table S2 Antibodies used in this study

Antibody target	Vendor	Cat#
β-actin	Cell Signaling Technology	4970
FLAG	Cell Signaling Technology	14793
PTBP1	abcam	ab5462
TPM4	abcam	ab181085
BCAP31	diagbio	db2421
MCCC2	proteintech	12117-1-1AP

Table S3 Quality control metrics of RNA-seq libraries in this study.

Sample_ID	Yield (Gb)	#Reads	% of >=Q30 Bases(PF)	Mean Quality Score (PF)	% of Mapping Rate
C33A2-1	10.57	75,633,316	97.92%	38.00	91.60
C33A2-2	10.18	72,871,048	97.93%	38.02	91.30
C33A2-3	10.42	74,599,338	97.66%	37.84	91.30
C33ANC-1	12.33	88,235,390	97.81%	37.94	91.80
C33ANC-2	11.52	82,463,320	97.91%	37.98	91.80
C33ANC-3	12.70	90,892,800	97.42%	37.72	90.40
CASKI2-1	9.80	70,135,968	97.24%	37.63	90.40
CASKI2-2	10.85	77,636,802	98.15%	38.14	91.10
CASKI2-3	11.52	82,483,170	97.15%	37.57	90.30
CASKINC-1	10.29	73,630,992	97.92%	38.01	92.00
CASKINC-2	9.40	67,318,328	98.28%	38.25	90.50
CASKINC-3	11.00	78,739,524	97.76%	37.92	91.30

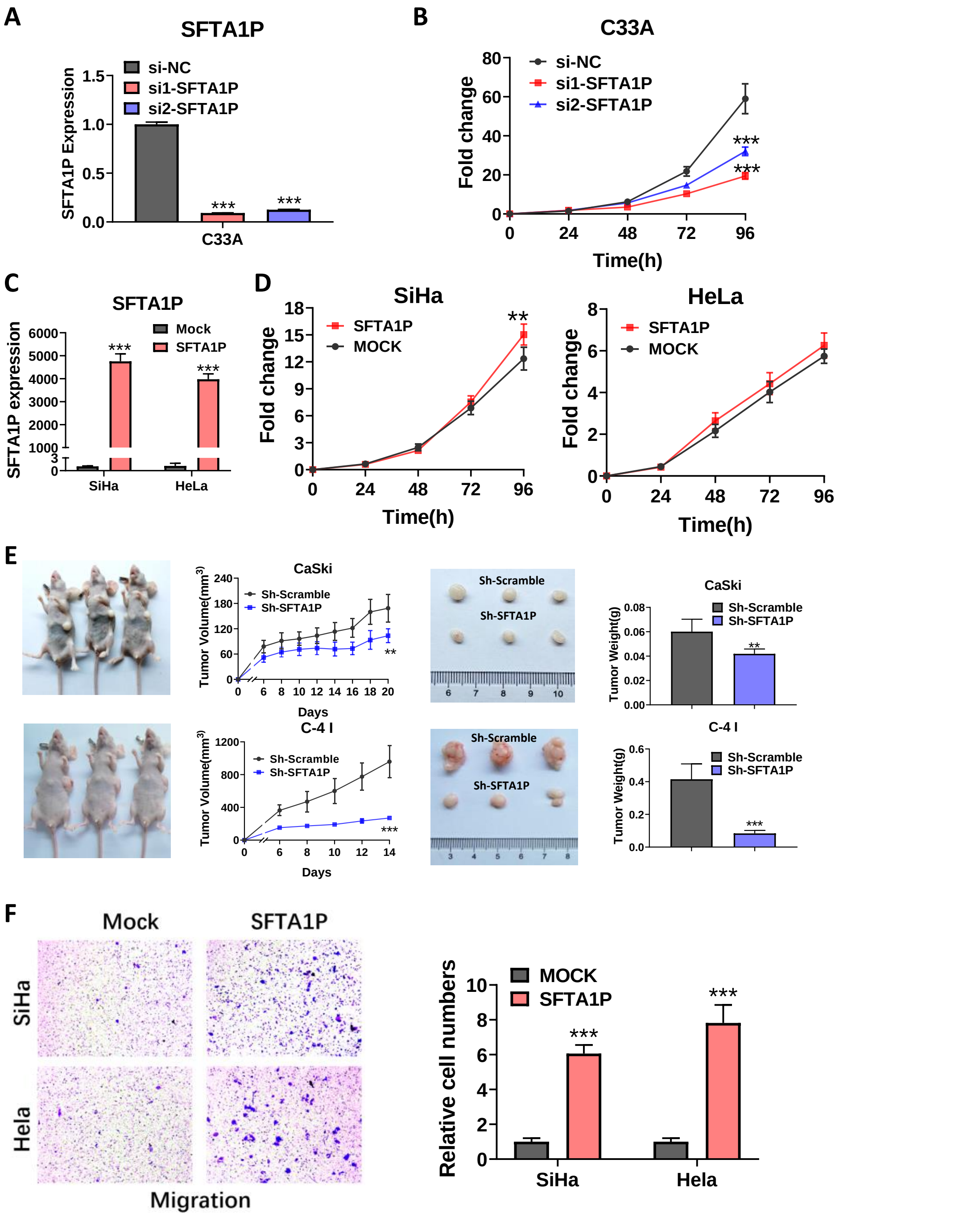

Figure S1 related to Figure 2 and 3. SFTA1P affects cervical cancer cell proliferation and migration. (**A**) Relative expression of SFTA1P in C33A transfected with siRNAs determined by qRT-PCR. (**B**) Growth curve of C33A transfected with SFTA1P siRNAs assessed by CCK8 assay. (**C**) Overexpression of SFTA1P in SiHa and HeLa cell lines. (**D**) Growth curve of SiHa and HeLa transfected with SFTA1P overexpression plasmid assessed by CCK8 assays. (**E**)Tumor volumes and weights of mice xenografts subcutaneously injected with CaSki or C-4 I sh-SFTA1P cells. Tumor volumes were normalized by the first measurement at day 7 after injection. Tumor weights were measured after mice were sacrificed 3 weeks after injection. (**F**) Migration of SiHa and HeLa cells transfected with SFTA1P overexpression plasmid. Data are shown as mean ± SEM. *$P < 0.05$; **$P < 0.01$; ***$P < 0.001$.

A SFTA1P RBPMAP

Protein: User_PTBP1

Position	Motif	Occurrence	Z-score	P-value
957	ucuu	acuguauauaagcaaaacagaagagucuuguuccaacagaaacucuggagcucc	2.455	7.04e-03
960	ucuu	guauauaagcaaaacagaagagucuuguuccaacagaaacucuggagcuccgug	1.893	2.92e-02
975	ucuu	agaagagucuuguuccaacagaaacucuggagcuccgugggucuuucucuucuc	2.571	5.07e-03
991	ucuu	aacagaaacucuggagcuccgugggucuuucucuucucuuguaagaaguuccuu	3.562	1.84e-04
995	ucuu	gaaacucuggagcuccgugggucuuucucuucucuuguaagaaguuccuuuugu	2.589	4.81e-03
997	ucuu	aacucuggagcuccgugggucuuucucuucucuuguaagaaguuccuuuuguua	3.571	1.78e-04
1000	ucuu	ucuggagcuccgugggucuuucucuucucuuguaagaaguuccuuuuguuauug	2.589	4.81e-03
1002	ucuu	uggagcuccgugggucuuucucuucucuuguaagaaguuccuuuuguuauugcc	3.571	1.78e-04
1015	ucuu	gucuuucucuucucuuguaagaaguuccuuuuguuauugccaucuucgcuuugc	2.661	3.90e-03
1016	ucuu	ucuuucucuucucuuguaagaaguuccuuuuguuauugccaucuucgcuuugcu	2.661	3.90e-03
1018	ucuu	uuucucuucucuuguaagaaguuccuuuuguuauugccaucuucgcuuugcugg	2.589	4.81e-03
1021	ucuu	cucuucucuuguaagaaguuccuuuuguuauugccaucuucgcuuugcuggaaa	2.589	4.81e-03
1024	ucuu	uucucuuguaagaaguuccuuuuguuauugccaucuucgcuuugcuggaaaugu	2.446	7.22e-03
1032	ucuu	uaagaaguuccuuuuguuauugccaucuucgcuuugcuggaaaugucaagcaaa	2.714	3.32e-03
1037	ucuu	aguuccuuuuguuauugccaucuucgcuuugcuggaaaugucaagcaaauuaug	2.161	1.53e-02
1151	ucuu	ucccuuuuuuuuucaaauggcaccagcuuuuucagcucucuuauuuuuuccuua	2.170	1.50e-02
1153	ucuu	ccuuuuuuuuucaaauggcaccagcuuuuucagcucucuuauuuuuuccuuaag	2.170	1.50e-02
1154	ucuu	cuuuuuuuuucaaauggcaccagcuuuuucagcucucuuauuuuuuccuuaagu	2.170	1.50e-02
1162	ucuu	uucaaauggcaccagcuuuuucagcucucuuauuuuuuccuuaaguagcauuua	2.170	1.50e-02
1164	ucuu	caaauggcaccagcuuuuucagcucucuuauuuuuuccuuaaguagcauuuauu	2.732	3.15e-03
1167	ucuu	auggcaccagcuuuuucagcucucuuauuuuuuccuuaaguagcauuuauuccu	2.170	1.50e-02
1169	ucuu	ggcaccagcuuuuucagcucucuuauuuuuuccuuaaguagcauuuauuccuaa	2.170	1.50e-02
1170	ucuu	gcaccagcuuuuucagcucucuuauuuuuuccuuaaguagcauuuauuccuaag	2.170	1.50e-02
1171	ucuu	caccagcuuuuucagcucucuuauuuuuuccuuaaguagcauuuauuccuaagg	2.170	1.50e-02
1174	ucuu	cagcuuuuucagcucucuuauuuuuuccuuaaguagcauuuauuccuaagguag	2.170	1.50e-02
1175	ucuu	agcuuuuucagcucucuuauuuuuuccuuaaguagcauuuauuccuaagguagg	2.170	1.50e-02
1189	ucuu	ucuuauuuuuuccuuaaguagcauuuauuccuaagguaggcaggguauuuccua	2.170	1.50e-02
1209	ucuu	gcauuuauuccuaagguaggcaggguauuuccuaguaagcauacuuucuuaaga	2.143	1.61e-02
1213	ucuu	uuauuccuaagguaggcaggguauuuccuaguaagcauacuuucuuaagacgga	2.143	1.61e-02
1226	ucuu	aggcaggguauuuccuaguaagcauacuuucuuaagacggaggccauuugguuc	2.036	2.09e-02
1230	ucuu	aggguauuuccuaguaagcauacuuucuuaagacggaggccauuugguuccugg	2.661	3.90e-03
1253	ucuu	uuucuuaagacggaggccauuugguuccugggagaauaggcagccccacacuuu	1.893	2.92e-02
1508	ucuu	ccuaagaauccuggcccuucuccacucuccaccaugcaggacaaacaucuucuc	2.143	1.61e-02
1530	ucuu	cacucuccaccaugcaggacaaacaucuucucaagcagucaacguagaaugcuu	2.589	4.81e-03
1533	ucuu	ucuccaccaugcaggacaaacaucuucucaagcagucaacguagaaugcuuggg	2.036	2.09e-02
1555	ucuu	ucuucucaagcagucaacguagaaugcuugggaaauagucauaauuacccacau	2.036	2.09e-02
1613	ucuu	uaauuaauagaugguaauuaauugauccuugaugugauguucuuuugcauauuu	2.143	1.61e-02
1614	ucuu	aauuaauagaugguaauuaauugauccuugaugugauguucuuuugcauauuuc	2.143	1.61e-02
1625	ucuu	gguaauuaauugauccuugaugugauguucuuuugcauauuuccuucauucuaa	2.170	1.50e-02
1628	ucuu	aauuaauugauccuugaugugauguucuuuugcauauuuccuucauucuaaagu	2.732	3.15e-03
1630	ucuu	uuaauugauccuugaugugauguucuuuugcauauuuccuucauucuaaaguug	2.170	1.50e-02
1637	ucuu	auccuugaugugauguucuuuugcauauuuccuucauucuaaaguuguucccug	2.170	1.50e-02
1641	ucuu	uugaugugauguucuuuugcauauuuccuucauucuaaaguuguucccuggccg	2.170	1.50e-02
1642	ucuu	ugaugugauguucuuuugcauauuuccuucauucuaaaguuguucccuggccgg	2.170	1.50e-02
1645	ucuu	ugugauguucuuuugcauauuuccuucauucuaaaguuguucccuggccgggag	2.170	1.50e-02
1649	ucuu	auguucuuuugcauauuuccuucauucuaaaguuguucccuggccgggagcguu	2.170	1.50e-02
2180	ucuu	ucagggaacagcaaauugaggauuuacuuaucuaggacuugaauuccuucuuug	2.143	1.61e-02
2185	ucuu	gaacagcaaauugaggauuuacuuaucuaggacuugaauuccuucuuugggacc	2.143	1.61e-02
2191	ucuu	caaauugaggauuuacuuaucuaggacuugaauuccuucuuugggaccaaguua	2.143	1.61e-02
2199	ucuu	ggauuuacuuaucuaggacuugaauuccuucuuugggaccaaguuaauaaaaga	2.143	1.61e-02
2200	ucuu	gauuuacuuaucuaggacuugaauuccuucuuugggaccaaguuaauaaaagac	2.143	1.61e-02
2203	ucuu	uuacuuaucuaggacuugaauuccuucuuugggaccaaguuaauaaaagaccaa	2.696	3.51e-03
2289	ucuu	ucuguagacagggcugcacguaucggcuuuguuugacuucucuuuucucaguua	2.170	1.50e-02
2293	ucuu	uagacagggcugcacguaucggcuuuguuugacuucucuuuucucaguuaacau	2.161	1.53e-02
2299	ucuu	gggcugcacguaucggcuuuguuugacuucucuuuucucaguuaacaucucaga	2.170	1.50e-02
2302	ucuu	cugcacguaucggcuuuguuugacuucucuuuucucaguuaacaucucagagcu	2.170	1.50e-02
2304	ucuu	gcacguaucggcuuuguuugacuucucuuuucucaguuaacaucucagagcuag	2.723	3.23e-03
2306	ucuu	acguaucggcuuuguuugacuucucuuuucucaguuaacaucucagagcuagaa	2.170	1.50e-02
2309	ucuu	uaucggcuuuguuugacuucucuuuucucaguuaacaucucagagcuagaacau	2.170	1.50e-02
2321	ucuu	uugacuucucuuuucucaguuaacaucucagagcuagaacauuccacauucccc	2.143	1.61e-02
2518	ucuu	aauugugcaauggaauaaaauccacacuuuagauucuugcaacuguaucauaug	1.893	2.92e-02
2527	ucuu	auggaauaaaauccacacuuuagauucuugcaacuguaucauauguaauaguau	2.455	7.04e-03
2540	ucuu	cacacuuuagauucuugcaacuguaucauauguaauaguaucacuuuuucuaca	2.161	1.53e-02

B

Protein IDs	Unique peptides
sp|P14618|PKM2_HUMAN	14
sp|Q9HCC0|MCCC2_HUMAN	7
sp|P26599|PTBP1_HUMAN	6
sp|P14866|HNRPL_HUMAN	4
sp|P36578|RL4_HUMAN	3
sp|P25705|ATPA_HUMAN	3
sp|P09874|PARP1_HUMAN	3
sp|P81605|DCD_HUMAN	2
sp|P12273|PIP_HUMAN	2
sp|P35637|FUS_HUMAN	2
sp|P60660|MYL6_HUMAN	2
sp|P61978|HNRPK_HUMAN	2
sp|P05166|PCCB_HUMAN	2
sp|O95758|PTBP3_HUMAN	2
sp|P10809|CH60_HUMAN	2
sp|Q02413|DSG1_HUMAN	2

C

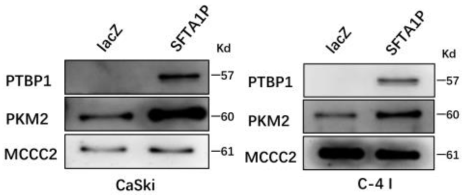

Figure S2 related to Figure 4. SFTA1P can bind to PTBP1. (**A**) Prediction of binding sites of PTBP1 with SFTA1P mRNA, including positions, motifs, occurrences, Z-scores, and P-values by RBPMAP. (**B**) Potential proteins that bind to SFTA1P as identified using mass spectrometry analysis. (**C**) Western blot to determine the specific interaction between SFTA1P and PTBP1 in CaSki and C-4 I lysates.

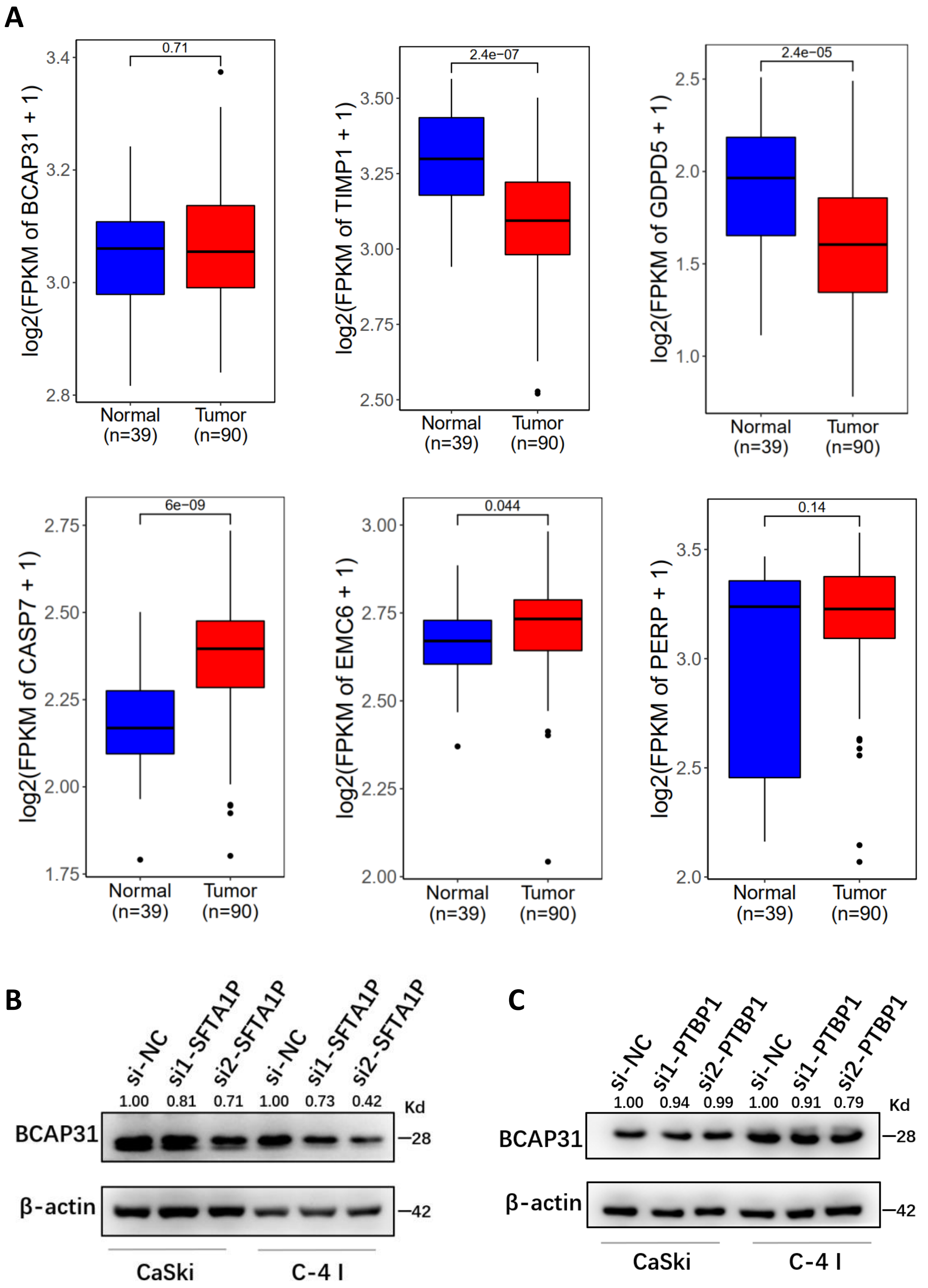

Figure S3 related to Figure 5. Downstream genes expression in cervical cancer. (**A**) Relative mRNA expression BCAP31, TIMP1, GDPD5, CASP7, EMC6 and PERP in cervical cancer from our previous RNA-seq data. (**B**) Western blot analysis of BCAP31 in cervical cancer cells with SFTA1P knockdown. (**C**) Western blot analysis of BCAP31 in cervical cancer cells with PTBP1 knockdown. Data are shown as mean ± SEM. *P < 0.05; **P < 0.01; ***P < 0.001.

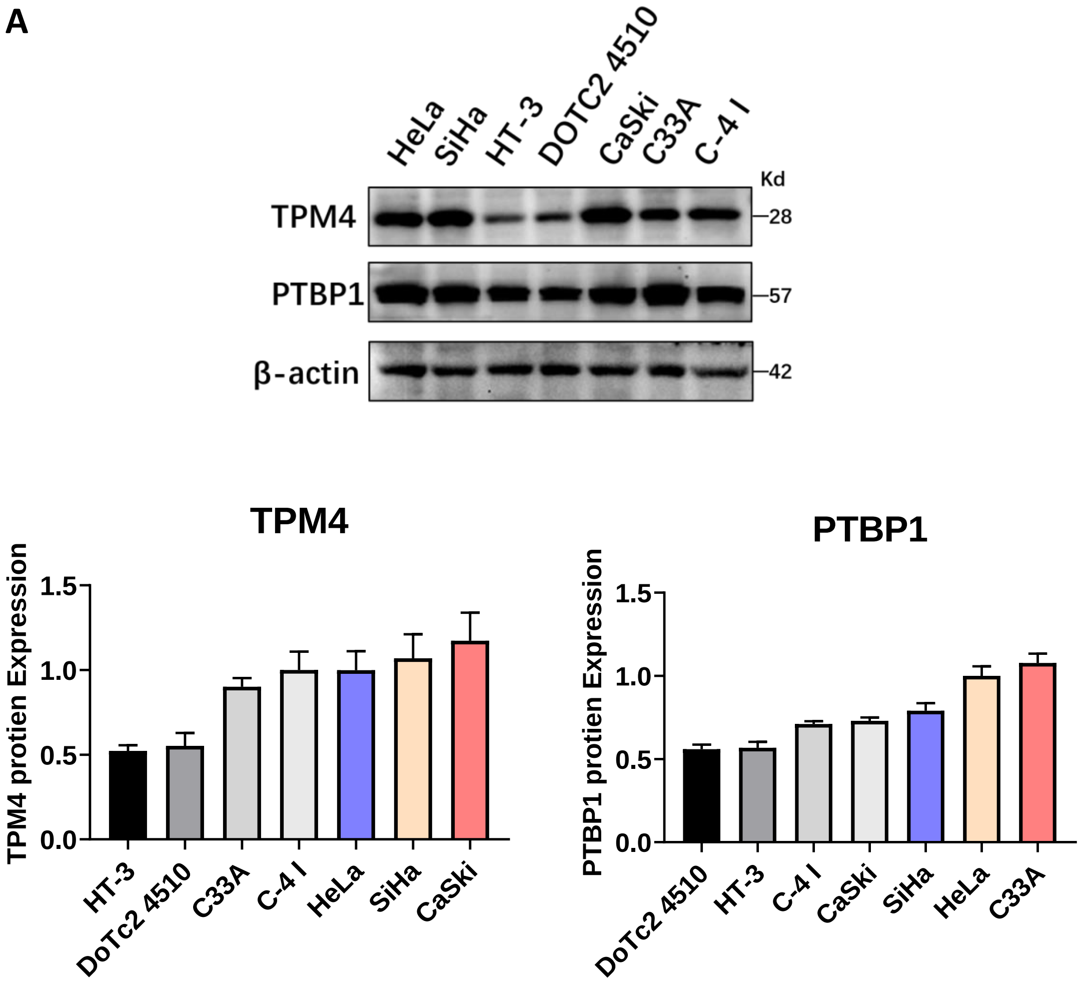

Figure S4 related to Figure 6. Basal expression of TPM4 and PTBP1 in cervical cell lines. (A) Relative protein expression levels of TPM4 and PTBP1 in different cervical cancer cell lines detected by Western blot analysis.

A

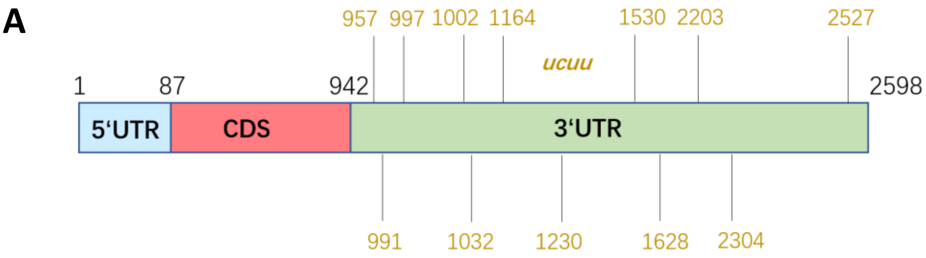

B

TPM4 RBPMAP

Protein: User_PTBP1

Position	Motif	Occurrence	Z-score	P-value
957	ucuu	acuguauauaagcaaaacagaagagucuuguuccaacagaaacucuggagcucc	2.446	7.22e-03
960	ucuu	guauauaagcaaaacagaagagucuuguuccaacagaaacucuggagcuccgug	1.884	2.98e-02
975	ucuu	agaagagucuuguuccaacagaaacucuggagcuccgugggucuuucucuucuc	2.563	5.19e-03
991	ucuu	aacagaaacucuggagcuccgugggucuuucucuucucuuguaagaaguuccuu	3.554	1.90e-04
995	ucuu	gaaacucuggagcuccgugggucuuucucuucucuuguaagaaguuccuuuugu	2.580	4.94e-03
997	ucuu	aacucuggagcuccgugggucuuucucuucucuuguaagaaguuccuuuuguua	3.562	1.84e-04
1000	ucuu	ucuggagcuccgugggucuuucucuucucuuguaagaaguuccuuuuguuauug	2.580	4.94e-03
1002	ucuu	uggagcuccgugggucuuucucuucucuuguaagaaguuccuuuuguuauugcc	3.562	1.84e-04
1015	ucuu	gucuuucucuucucuuguaagaaguuccuuuuguuauugccaucuucgcuuugc	2.652	4.00e-03
1016	ucuu	ucuuucucuucucuuguaagaaguuccuuuuguuauugccaucuucgcuuugcu	2.652	4.00e-03
1018	ucuu	uuucucuucucuuguaagaaguuccuuuuguuauugccaucuucgcuuugcugg	2.580	4.94e-03
1021	ucuu	cucuucucuuguaagaaguuccuuuuguuauugccaucuucgcuuugcuggaaa	2.580	4.94e-03
1024	ucuu	uucucuuguaagaaguuccuuuuguuauugccaucuucgcuuugcuggaaaugu	2.438	7.38e-03
1032	ucuu	uaagaaguuccuuuuguuauugccaucuucgcuuugcuggaaaugucaagcaaa	2.705	3.42e-03
1037	ucuu	aguuccuuuuguuauugccaucuucgcuuugcuggaaaugucaagcaaauuaug	2.152	1.57e-02
1151	ucuu	ucccuuuuuuuuucaaauggcaccagcuuuuucagcucucuuauuuuuuccuua	2.161	1.53e-02
1153	ucuu	ccuuuuuuuuucaaauggcaccagcuuuuucagcucucuuauuuuuuccuuaag	2.161	1.53e-02
1154	ucuu	cuuuuuuuuucaaauggcaccagcuuuuucagcucucuuauuuuuuccuuaagu	2.161	1.53e-02
1162	ucuu	uucaaauggcaccagcuuuuucagcucucuuauuuuuuccuuaaguagcauuua	2.161	1.53e-02
1164	ucuu	caaauggcaccagcuuuuucagcucucuuauuuuuuccuuaaguagcauuuauu	2.723	3.23e-03
1167	ucuu	auggcaccagcuuuuucagcucucuuauuuuuuccuuaaguagcauuuauuccu	2.161	1.53e-02
1169	ucuu	ggcaccagcuuuuucagcucucuuauuuuuuccuuaaguagcauuuauuccuaa	2.161	1.53e-02
1170	ucuu	gcaccagcuuuuucagcucucuuauuuuuuccuuaaguagcauuuauuccuaag	2.161	1.53e-02
1171	ucuu	caccagcuuuuucagcucucuuauuuuuuccuuaaguagcauuuauuccuaagg	2.161	1.53e-02
1174	ucuu	cagcuuuuucagcucucuuauuuuuuccuuaaguagcauuuauuccuaagguag	2.161	1.53e-02
1175	ucuu	agcuuuuucagcucucuuauuuuuuccuuaaguagcauuuauuccuaagguagg	2.161	1.53e-02
1189	ucuu	ucuuauuuuuuccuuaaguagcauuuauuccuaagguaggcaggguauuuccua	2.161	1.53e-02
1209	ucuu	gcauuuauuccuaagguaggcaggguauuuccuaguaagcauacuuucuuaaga	2.134	1.64e-02
1213	ucuu	uuauuccuaagguaggcaggguauuuccuaguaagcauacuuucuuaagacgga	2.134	1.64e-02
1226	ucuu	aggcaggguauuuccuaguaagcauacuuucuuaagacggaggccauuugguuc	2.027	2.13e-02
1230	ucuu	aggguauuuccuaguaagcauacuuucuuaagacggaggccauuugguuccugg	2.652	4.00e-03
1253	ucuu	uuucuuaagacggaggccauuugguuccugggagaauaggcagccccacacuuu	1.884	2.98e-02
1508	ucuu	ccuaagaauccuggcccuucuccacucuccaccaugcaggacaaacaucuucuc	2.134	1.64e-02
1530	ucuu	cacucuccaccaugcaggacaaacaucuucucaagcagucaacguagaaugcuu	2.580	4.94e-03
1533	ucuu	ucuccaccaugcaggacaaacaucuucucaagcagucaacguagaaugcuuggg	2.027	2.13e-02
1555	ucuu	ucuucucaagcagucaacguagaaugcuugggaaauagucauaauuacccacau	2.027	2.13e-02
1613	ucuu	uaauuaauagaugguaauuaauugauccuugaugugauguucuuuugcauauuu	2.134	1.64e-02
1614	ucuu	aauuaauagaugguaauuaauugauccuugaugugauguucuuuugcauauuuc	2.134	1.64e-02
1625	ucuu	gguaauuaauugauccuugaugugauguucuuuugcauauuuccuucauucuaa	2.161	1.53e-02
1628	ucuu	aauuaauugauccuugaugugauguucuuuugcauauuuccuucauucuaaagu	2.723	3.23e-03
1630	ucuu	uuaauugauccuugaugugauguucuuuugcauauuuccuucauucuaaaguug	2.161	1.53e-02
1637	ucuu	auccuugaugugauguucuuuugcauauuuccuucauucuaaaguuguucccug	2.161	1.53e-02
1641	ucuu	uugaugugauguucuuuugcauauuuccuucauucuaaaguuguucccuggccg	2.161	1.53e-02
1642	ucuu	ugaugugauguucuuuugcauauuuccuucauucuaaaguuguucccuggccgg	2.161	1.53e-02
1645	ucuu	ugugauguucuuuugcauauuuccuucauucuaaaguuguucccuggccgggag	2.161	1.53e-02
1649	ucuu	auguucuuuugcauauuuccuucauucuaaaguuguucccuggccgggagcguu	2.161	1.53e-02
2180	ucuu	ucagggaacagcaaauugaggauuuacuuaucuaggacuugaauuccuucuuug	2.134	1.64e-02
2185	ucuu	gaacagcaaauugaggauuuacuuaucuaggacuugaauuccuucuuugggacc	2.134	1.64e-02
2191	ucuu	caaauugaggauuuacuuaucuaggacuugaauuccuucuuugggaccaaguua	2.134	1.64e-02
2199	ucuu	ggauuuacuuaucuaggacuugaauuccuucuuugggaccaaguuaauaaaaga	2.134	1.64e-02
2200	ucuu	gauuuacuuaucuaggacuugaauuccuucuuugggaccaaguuaauaaaagac	2.134	1.64e-02
2203	ucuu	uuacuuaucuaggacuugaauuccuucuuugggaccaaguuaauaaaagaccaa	2.688	3.59e-03
2289	ucuu	ucuguagacagggcugcacguaucggcuuuguuugacuucucuuuucucaguua	2.161	1.53e-02
2293	ucuu	uagacagggcugcacguaucggcuuuguuugacuucucuuuucucaguuaacau	2.152	1.57e-02
2299	ucuu	gggcugcacguaucggcuuuguuugacuucucuuuucucaguuaacaucucaga	2.161	1.53e-02
2302	ucuu	cugcacguaucggcuuuguuugacuucucuuuucucaguuaacaucucagagcu	2.161	1.53e-02
2304	ucuu	gcacguaucggcuuuguuugacuucucuuuucucaguuaacaucucagagcuag	2.714	3.32e-03
2306	ucuu	acguaucggcuuuguuugacuucucuuuucucaguuaacaucucagagcuagaa	2.161	1.53e-02
2309	ucuu	uaucggcuuuguuugacuucucuuuucucaguuaacaucucagagcuagaacau	2.161	1.53e-02
2321	ucuu	uugacuucucuuuucucaguuaacaucucagagcuagaacauuccacauucccc	2.134	1.64e-02
2518	ucuu	aauugugcaauggaauaaaauccacacuuuagauucuugcaacuguaucauaug	1.884	2.98e-02
2527	ucuu	auggaauaaaauccacacuuuagauucuugcaacuguaucauauguaauaguau	2.446	7.22e-03
2540	ucuu	cacacuuuagauucuugcaacuguaucauauguaauaguaucacuuuuucuaca	2.152	1.57e-02

Figure S5 related to Figure 7. TPM4 mRNA can bind to PTBP1. (**A**) Schematic of PTBP1 binding sites (UCUU) within the 3′UTR of TPM4 mRNA, predicted by RBPMAP. (**B**) Prediction of the binding sites of PTBP1 with TPM4 mRNA, including positions, motifs, occurrences, Z-scores, and P-values by RBPMAP.

03/30/2016 11:08:16

BOX CONTENTS

SHIPMENT#: SOJ31463

Box No: 1 Of 2

ATCC™

10801 University Boulevard
Manassas, Virginia 20110-2209 USA
703-365-2700 FAX: 703-365-2750
EMAIL: sales@atcc.org

ATCC 10801 University Blvd. Manassas, VA 20110-2209 USA
EMERGENCY RESPONSE: Chemtrec (800) 424-9300 or (202) 483-7616

BILL-TO:
7513
National Institutes of Healt
Commercial Accts Branch/AP
MSC 8500 Ste 4B-432
2115 E Jefferson St
Rockville, MD 20852
USA

ATTN: Accounts, Payable
TEL#: 301-496-6088 FAX#: 301-496-3588

SHIP-TO:
00181061
National Institutes of Health
BLDG C ROOM 311W
WEI ZHENG/NI SIMA/3552
9800 Medical Center Dr
Rockville, MD 20850
USA

ATTN: Zheng, Wei
TEL#: 301-451-8682 FAX#: 999
CUSTOMER PO: 16-003552

SOLD TO: 7513<br>ORDER DATE 03/30/16<br>SALESPERSON: 100	DAP: Rockville<br>BOL:	**SHIPPED VIA** Custom Courier<br>**SHIP DATE:** 03/30/16	FREIGHT LIST<br>NIH only

#	BSL	ITEM NUMBER		UM	QTY SHIP	QTY B.O.	DESCRIPTION		LOT #	REF #
003	2	CRL-1550	FZ	EA	1	0	Ca Ski; Cervical Carcinoma; Human (Homo sapiens) Attention: Zeng, Wei		61978337	
005	2	CRL-3216	FZ	EA	1	0	293T; Embryonic Kidney Cells; Human (Homo sapiens) Attention: Zeng, Wei		62729596	
006	1	PCS-200-010	FZ	EA	1	0	Primary Epidermal Keratinocytes; Normal, Human, Neonatal Attention: Zeng, Wei		63176925	
004	1	HTB-31	FZ	EA	1	0	C-33 A; Cervical Carcinoma; Human (Homo sapiens) Attention: Zeng, Wei		63596879	
002	1	PCS-100-010	FZ	EA	1	0	Primary Umbilical Vein Endothelial Cells; Normal, Human Attention: Zeng, Wei<br>TOTAL QTY. THIS BOX 5		62245802	

Page # 1

Figure S6. ATCC purchase history of CaSki and C33A cell lines.

ATCC ORDER FORM

ATCC DISTRIBUTOR

Beijing Zhongyuan Ltd.
http://www.sinozhongyuan.com
Email: office@sinozhongyuan.com

PRODUCTS ORDERED 订购产品列表

ATCC Product No.<br>ATCC 产品号	Description<br>货物描述	Qty.<br>数量	Unit Price<br>单价	进口产品用途		
				用于什么实验	简单的实验流程描述	实验研究结果及用途
ATCC® HTB-111	人子宫内膜癌细胞株 AN3 CA	1	7803	用于研究癌症发生机制	将买回来的细胞进行转基因和药物干预，达到对细胞基因进行修饰，从而研究子宫内膜癌的发生机制研究	用以研究蛋白变化对子宫内膜癌的影响
ATCC® HTB-112	人子宫内膜癌细胞株 HEC-1-A	1	6103	用于研究癌症发生机制	将买回来的细胞进行转基因和药物干预，达到对细胞基因进行修饰，从而研究子宫内膜癌的发生机制研究	用以研究蛋白变化对子宫内膜癌的影响
ATCC® HTB-32	人宫颈癌细胞株HT-3	1	6103	用于研究癌症发生机制	将买回来的细胞进行转基因和药物干预，达到对细胞基因进行修饰，从而研究宫颈癌的发生机制研究	用以研究蛋白变化对宫颈癌的影响
ATCC® CRL-1594	人宫颈癌细胞株 C-4 I	1	7803	用于研究癌症发生机制	将买回来的细胞进行转基因和药物干预，达到对细胞基因进行修饰，从而研究宫颈癌的发生机制研究	用以研究蛋白变化对宫颈癌的影响
ATCC® CRL-7920	人宫颈癌细胞株DoTc2 4510	1	7803	用于研究癌症发生机制	将买回来的细胞进行转基因和药物干预，达到对细胞基因进行修饰，从而研究宫颈癌的发生机制研究	用以研究蛋白变化对宫颈癌的影响
30-2007	McCoy's 5A Medium	1				

【填表说明：上表中第一行进口产品用途，仅为举例说明，并不是模板。请订购单位根据进口产品的实验用途填写。若所订购多项产品用于一个实验用途，可合并填写】

PAYMENT INFORMATION 付款信息

When receiving this order, we will fax a confirmation and sales contract to you. Please finish the payment according to contract to below bank information.
收到订单后，我司会传真订单确认和进口代理协议给您。请按照合同要求付货款至我司下面银行。

开户行：中国建设银行北京分行花园路分理处
银行帐号：110010-285000-56034676
公司名称：北京中原合聚经贸有限公司
公司地址：北京市朝阳区东方东路 11 号

Thank you for your order. By submitting this order, you are accepting the terms of our Material Transfer Agreement, available at www.atcc.org and www.sinozhongyuan.com . Shipment is contingent upon

Figure S7. ATCC purchase history of HT-3, C-4 I and DOTC2 4510 cell lines.

ATCC™ ORDER FORM

ATCC DISTRIBUTOR

Beijing Zhongyuan Ltd.
http://www.sinozhongyuan.com
Email: office@sinozhongyuan.com

PRODUCTS ORDERED 订购产品列表

ATCC Product No. ATCC 产品号	Description 货物描述	Qty. 数量	Unit Price 单价	进口产品用途		
				用于什么实验	简单的实验流程描述	实验研究结果及用途
ATCC HTB-35	SiHa 细胞	1	CNY6103	用于细胞实验	研究 E6\E7 在宫颈癌 SiHa 细胞中的作用	研究病毒的变化对宫颈癌细胞的影响

【填表说明：上表中第一行进口产品用途，仅为举例说明，并不是模板。请订购单位根据进口产品的实验用途填写。若所订购多项产品用于一个实验用途，可合并填写】

PAYMENT INFORMATION 付款信息

When receiving this order, we will fax a confirmation and sales contract to you. Please finish the payment according to contract to below bank information.
收到订单后，我司会传真订单确认和进口代理协议给您。请按照合同要求付货款至我司下面银行。

开户行：中国建设银行北京分行花园路分理处
银行帐号：110010-285000-56034676
公司名称：北京中原合聚经贸有限公司
公司地址：北京市朝阳区东方东路 11 号

Thank you for your order. By submitting this order, you are accepting the terms of our Material Transfer Agreement, available at **www.atcc.org** and **www.sinozhongyuan.com** . Shipment is contingent upon confirmation of your customer account information and compliance with all applicable regulations and permit requirements.
感谢订购 ATCC 的产品。提交此订单，即证明您已经同意接受 ATCC 的 MTA 协议（您可以在 ATCC 和中原公司的网站上查看 MTA 相关条款）。在您的帐户信息确认无误、且所订产品符合中国海关政策的情况下，我公司会安排发货。

传真: 0086-10-84415679 电话: 0086-10-84415678 email: atcc@sinozhongyuan.com

Figure S8. ATCC purchase history of SiHa.

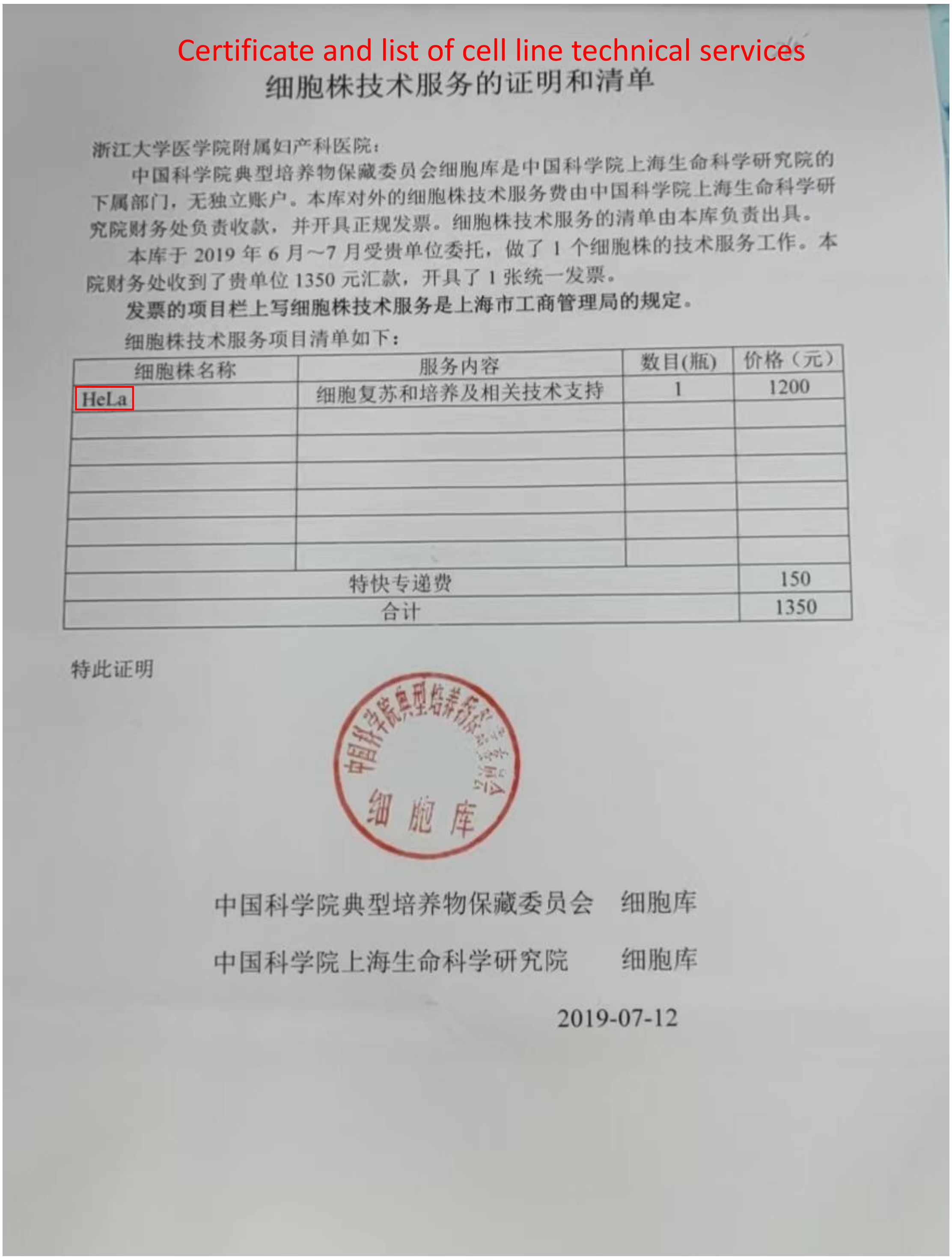

Certificate and list of cell line technical services

细胞株技术服务的证明和清单

浙江大学医学院附属妇产科医院：

中国科学院典型培养物保藏委员会细胞库是中国科学院上海生命科学研究院的下属部门，无独立账户。本库对外的细胞株技术服务费由中国科学院上海生命科学研究院财务处负责收款，并开具正规发票。细胞株技术服务的清单由本库负责出具。

本库于 2019 年 6 月～7 月受贵单位委托，做了 1 个细胞株的技术服务工作。本院财务处收到了贵单位 1350 元汇款，开具了 1 张统一发票。

发票的项目栏上写细胞株技术服务是上海市工商管理局的规定。

细胞株技术服务项目清单如下：

细胞株名称	服务内容	数目(瓶)	价格（元）
HeLa	细胞复苏和培养及相关技术支持	1	1200
特快专递费			150
合计			1350

特此证明

中国科学院典型培养物保藏委员会　细胞库

中国科学院上海生命科学研究院　　细胞库

2019-07-12

Figure S9. Cell line authentication data of HeLa.
