# Supplementary figures and images for: LncRNA SFTA1P promotes cervical cancer progression by interaction with PTBP1 to facilitate TPM4 mRNA degradation
Source: Cell Death Dis. 2022 Nov 7;13(11):936. doi: 10.1038/s41419-022-05359-7 (PMC9640654; doi:10.1038/s41419-022-05359-7)

**Fig.4D**

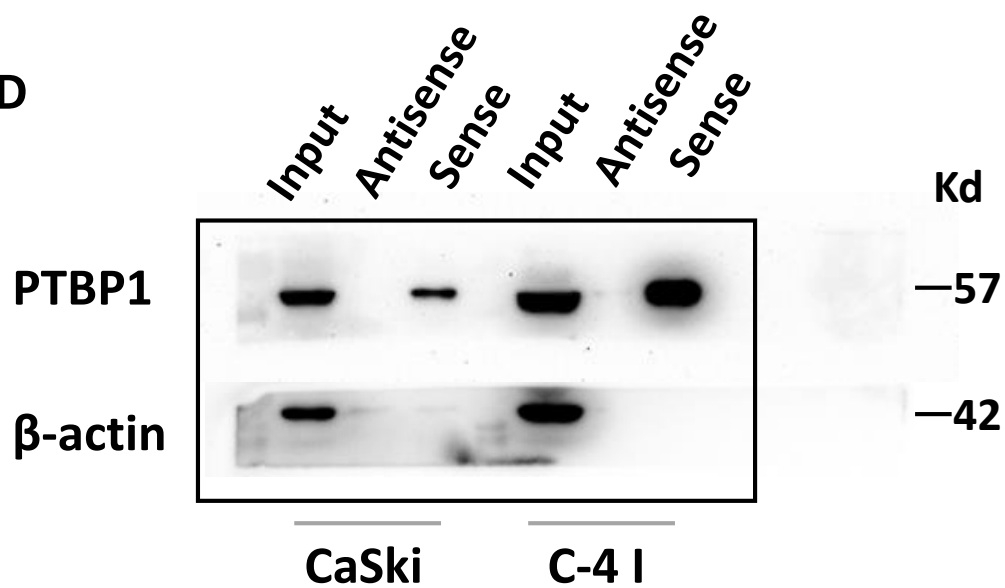

**Fig.4G**

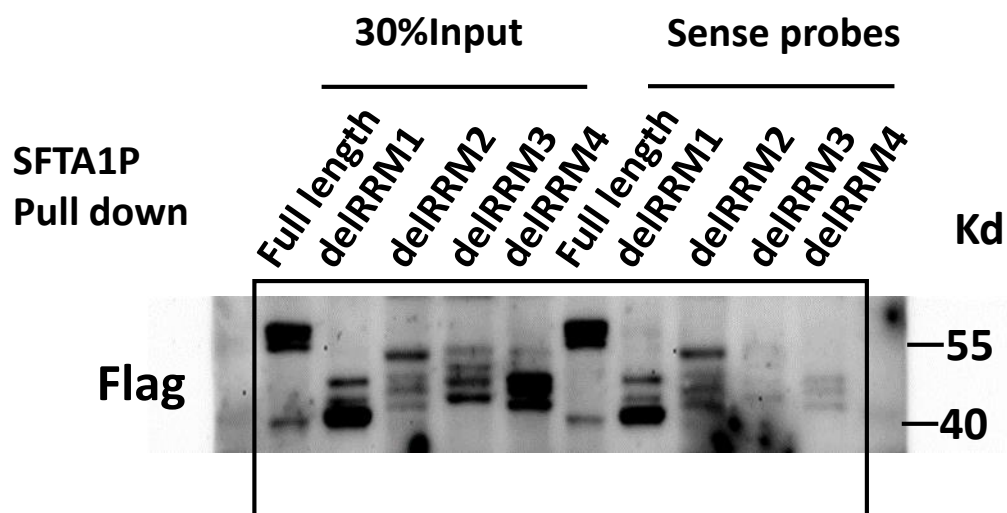

Fig.5D

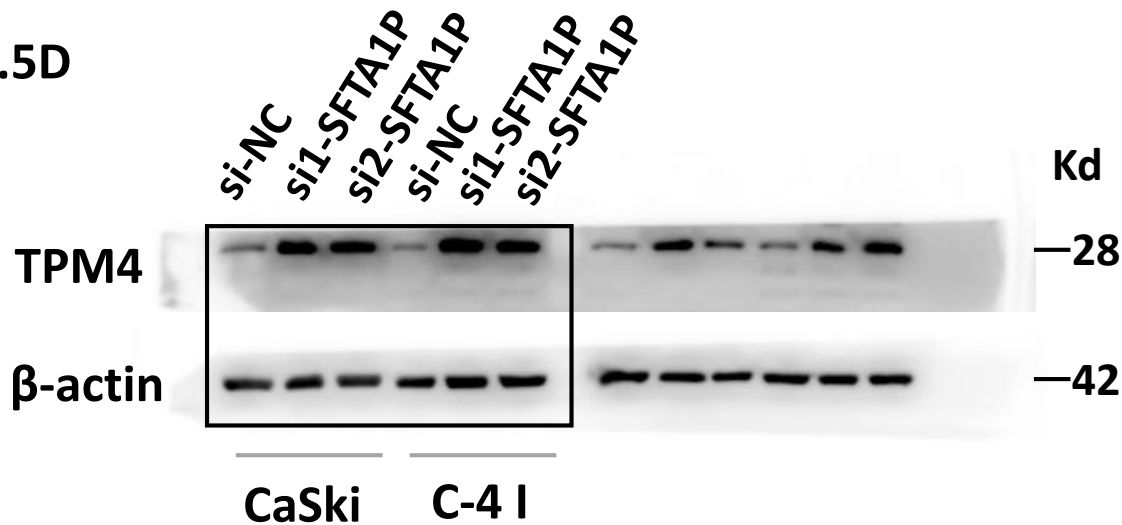

Fig.5F

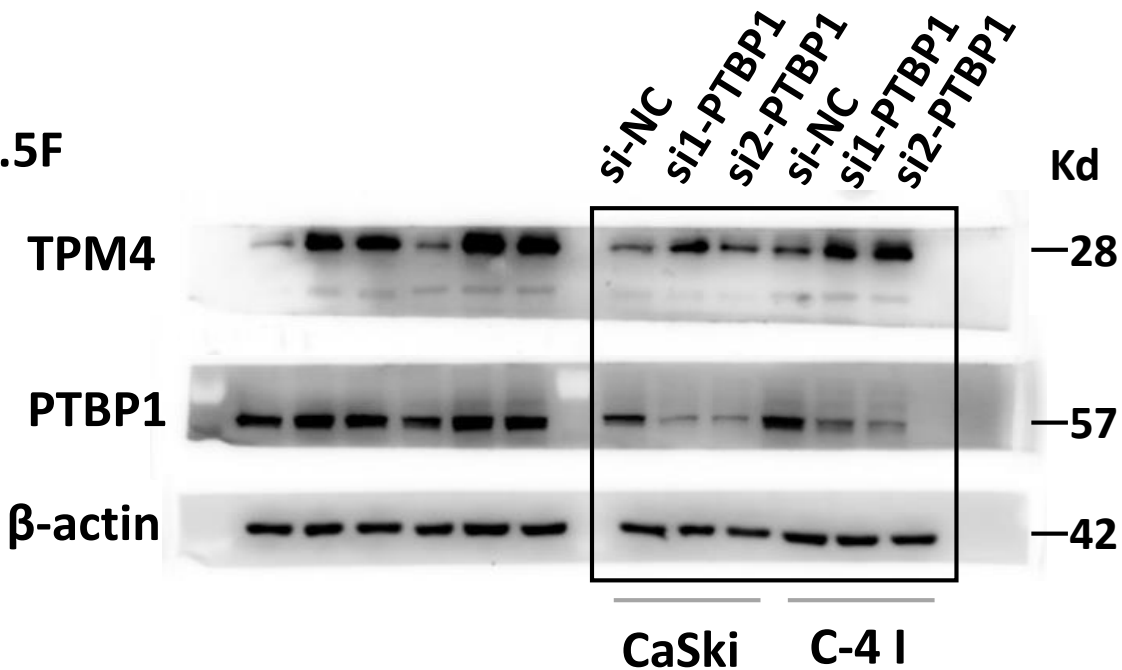

Fig.6B

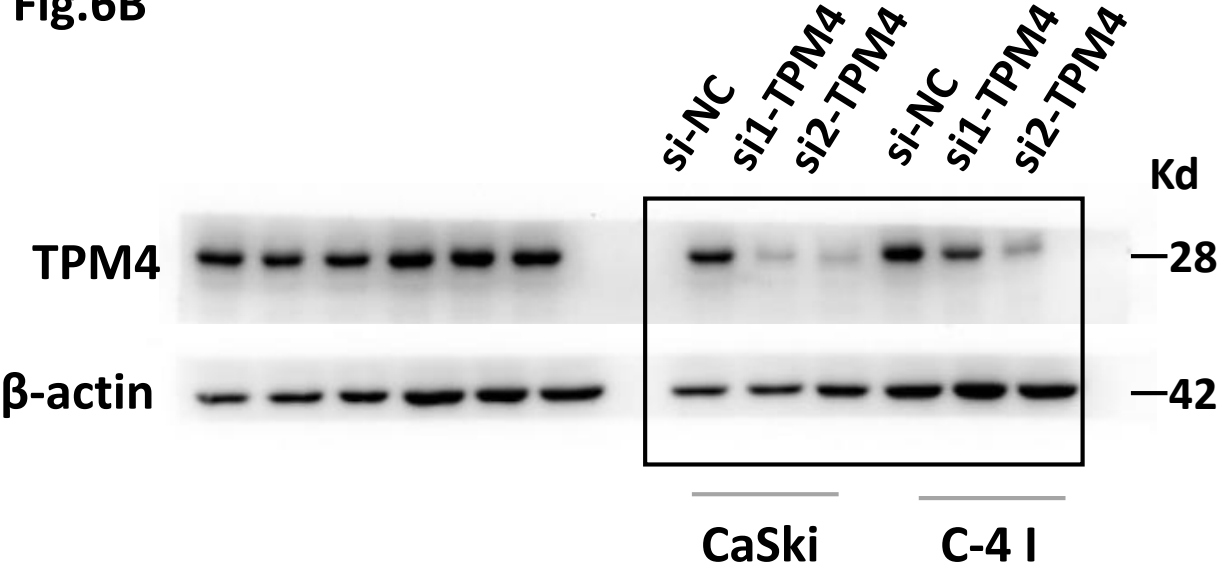

Fig.7A

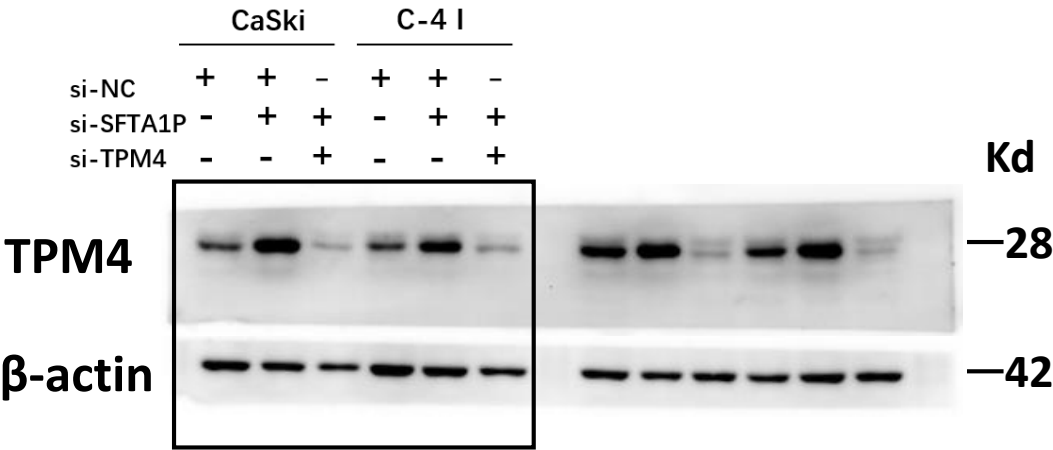

Fig. 7C

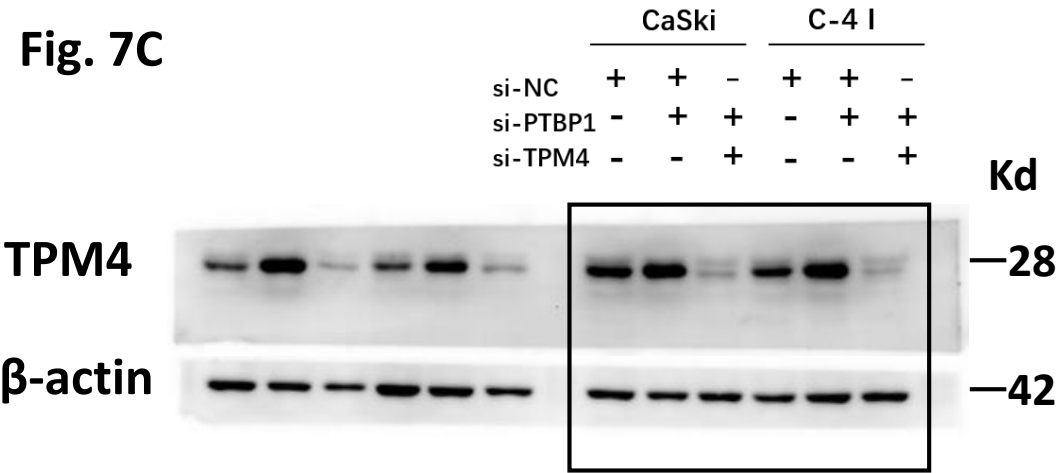

Fig. 7F

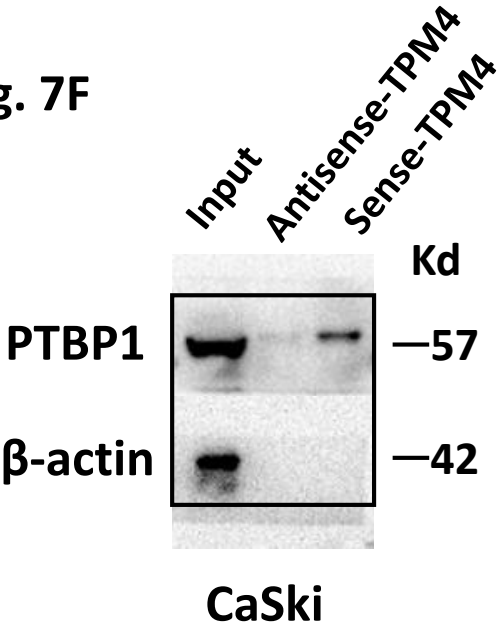

Fig.S2C

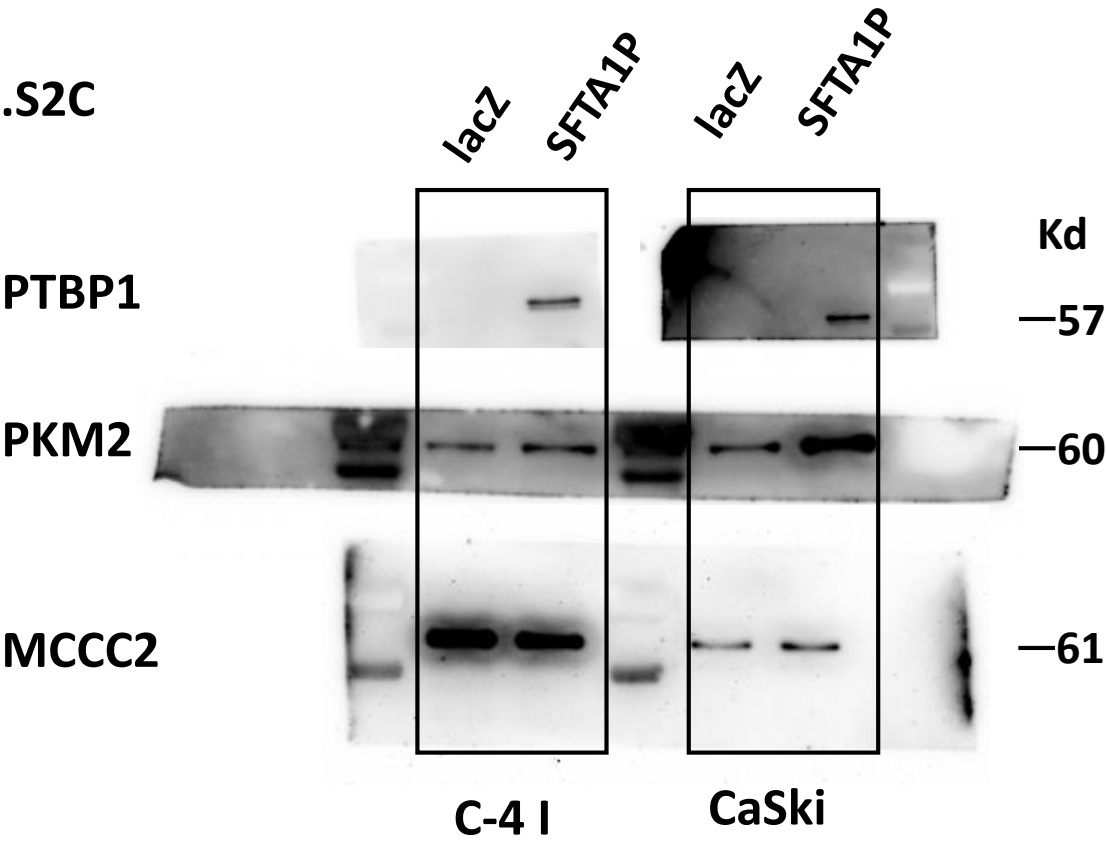

Fig.S3B

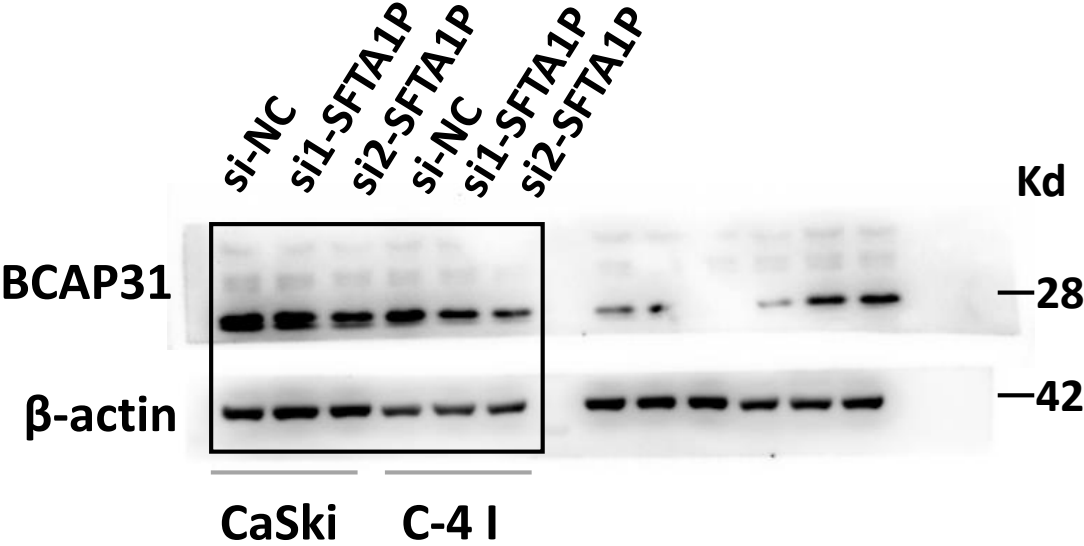

Fig.S3C

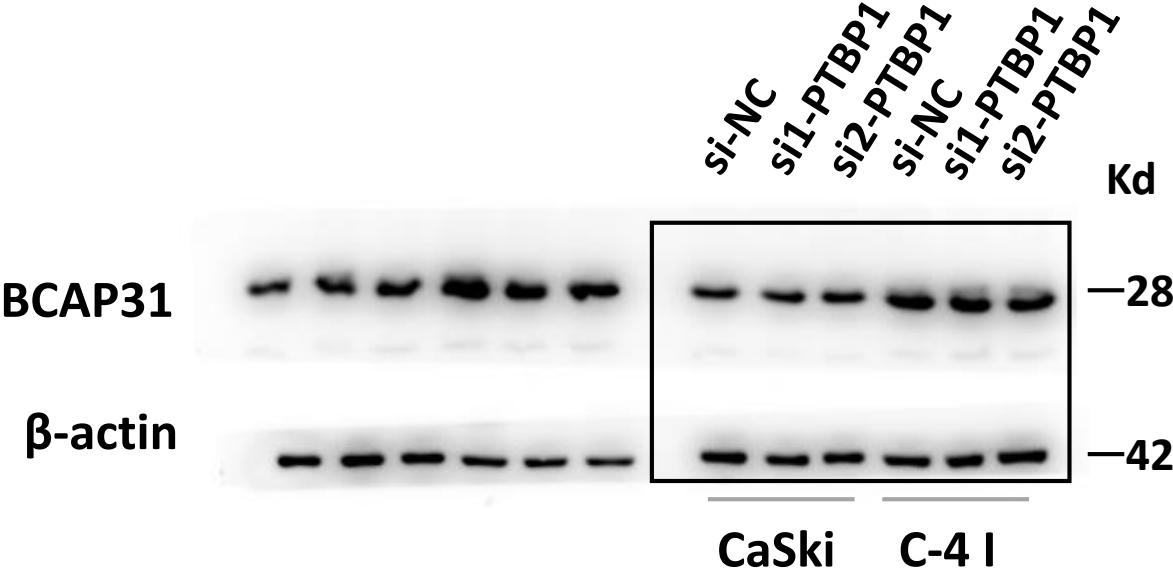

**Fig.S4A**

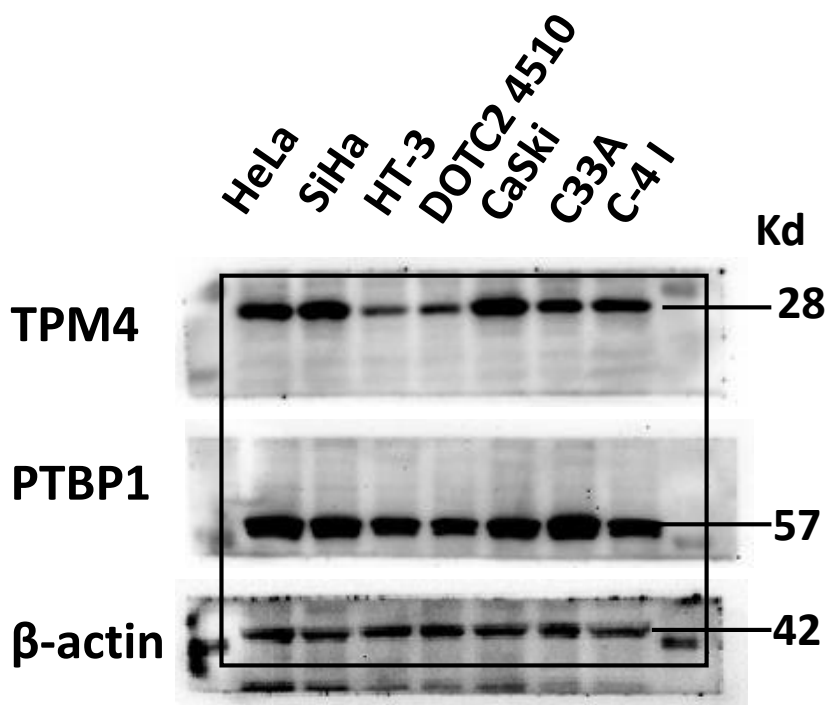

Supplement: Supplementary file 2 — Original Data File [file 41419_2022_5359_MOESM2_ESM.pdf]
